# Epidemiological and molecular characterisation of flea infestations in dogs and cats in mainland Portugal

**DOI:** 10.1186/s13071-025-06904-x

**Published:** 2025-07-06

**Authors:** André Pereira, Adrian Cruz, Teresa Novo, FleaPT group, Ana Cardoso, Ana Oliva, Ana Róis, Ana Saragoça, André Silva, Andreia Pinto, Aniana Correia, Bruno Almeida, Gonçalo Lixa, Guida Brito, Hugo Vilhena, José Chaves, Karine Torres, Luís Martins, Madalena Lemos, Margarida Câmara, Miguel Almas, Patrícia Cachola, Paulo Afonso, Ricardo Dias, Rita Calouro, Ruth Gomes, Sabrina Rodrigues, Sara Lang, Sofia Piteira, Telma Gomes, Carla Maia

**Affiliations:** 1 Research in Veterinary Medicine (I-MVET), Faculty of Veterinary Medicine, Lusófona University-Lisbon University Centre, Campo Grande 376, 1749-024 Lisbon, Portugal; 2 Animal and Veterinary Research Center (CECAV), Faculty of Veterinary Medicine, Lusófona University-Lisbon University Centre, Campo Grande 376, 1749-024 Lisbon, Portugal; 3 Superior School of Health, Protection and Animal Welfare, Polytechnic Institute of Lusophony, Campo Grande 400, 1700-098 Lisbon, Portugal; 4 Global Health and Tropical Medicine (GHTM), LA-REAL, Instituto de Higiene E Medicina Tropical (IHMT), Universidade NOVA de Lisboa, Rua da Junqueira No. 100, 1349-008 Lisbon, Portugal

**Keywords:** Cats, *Ctenocephalides felis felis*, Dogs, Flea Infestations, Portugal

## Abstract

**Background:**

Fleas are major ectoparasites of dogs and cats, with significant veterinary and public health implications. This study aimed to estimate the prevalence of flea infestation in dogs and cats in mainland Portugal, identify associated risk factors and perform morphological and molecular characterisation of flea specimens.

**Methods:**

A cross-sectional study was conducted from March 2022 to February 2023 in mainland Portugal. Dogs and cats were screened for flea infestations at veterinary clinics and shelters following World Association for the Advancement of Veterinary Parasitology guidelines. Fleas were morphologically identified to species level, and a subset was characterised molecularly via phylogenetic analysis of the cytochrome* c* oxidase subunit II gene (*cox2*) sequences. Epidemiological data were analysed through multivariate logistic regression models to identify possible risk factors associated with flea infestation.

**Results:**

A total of 1052 dogs and 1039 cats were examined, and flea infestation prevalence was determined to be 33.6% and 36.5%, respectively. *Ctenocephalides felis* was the predominant flea species in both hosts, accounting for 85.7% of fleas in dogs and 98.8% in cats, with molecular data confirming the subspecies *C. felis felis*. Other species identified included *Ctenocephalides canis* (9.6% in dogs; 1.8% in cats), *Pulex irritans* (4.2% in dogs) and *Archaeopsylla erinacei maura* (0.8% in dogs). The absence of insecticide use was the strongest predictor of flea infestation in both hosts (dogs: adjusted odds ratio [aOR] 4.87; cats: aOR 4.02). In dogs, the risk of infestation was higher in spring, summer and autumn compared to winter (aOR 2.08–3.72), and lower in the Lisbon Metropolitan Area, Alentejo and Algarve compared to the North region (aOR 0.14–0.45). In cats, risk was reduced in non-northern regions (Lisbon, Alentejo, Centro; aOR 0.10–0.45) and in those cats with non-domestic outdoor lifestyles (aOR 0.19).

**Conclusions:**

Flea infestations are highly prevalent in dogs and cats across mainland Portugal, with *C. felis felis* as the dominant species. These findings provide valuable insights for the development of integrated, evidence-based strategies for flea control.

**Supplementary Information:**

The online version contains supplementary material available at 10.1186/s13071-025-06904-x.

## Background

Fleas (Order Siphonaptera) are periodic haematophagous ectoparasites that infest a wide range of avian and mammalian hosts, including dogs, cats and humans [1]. These insects are of major veterinary and public health concern, as they cause discomfort, trigger hypersensivity reactions such as flea allergy dermatitis (FAD) in susceptible hosts and act as competent vectors and/or intermediate hosts for several zoonotic pathogens, including *Bartonella* spp. (e.g. *B. clarridgeiae* and *B. henselae*), *Rickettsia felis* and *Dipylidium caninum* [1–9].

*Ctenocephalides felis*, the cat flea, is the most prevalent species infesting dogs and cats worldwide [7, 10–12]. Morphological and molecular studies have led to the recognition of three subspecies: *C. felis felis*, which has a cosmopolitan distribution, and *C. felis strongylus* and *C. felis damaranensis*, both of which are restricted to the African region [13]. Other flea species of medical concern that frequently infest companion animals include *Ctenocephalides canis* (dog flea) and *Pulex irritans* (human flea) [1, 2, 10]. Although less common, *Archaeopsylla erinacei, Echidnophaga gallinacea* and *Spilopsyllus cuniculi*, primarily ectoparasites of hedgehogs, poultry and rabbits, respectively, have also been reported in both dogs and cats [1, 4, 10, 14, 15].

Epidemiological studies indicate that flea prevalence and intensity are influenced by several factors, including climate, host factors and the preventive measures employed [5, 11, 14–16]. Optimal climatic conditions for flea survival and reproduction include temperatures ranging from 20 ºC to 30 ºC and relative humidity exceeding 70% [7, 12]. Host density and availability further contribute to flea infestation dynamics [2]. High infestation prevalence has been reported in southern European countries such as Spain, Italy, Greece and Cyprus, with *C. felis* being the predominant flea species in both dogs and cats [14–18].

Flea infestations remain frequent even though commercial ectoparasiticides are widely available [19]. This seems to be due to the high reproductive rate of fleas, their environmental adaptability and resilience, ubiquitous presence in human-animal environments and their decreased susceptibility to certain insecticides, including pyrethroids [2, 3, 5, 7, 10, 14]. These factors underscore the need for epidemiological surveillance to develop and refine evidence-based control strategies aimed at reducing flea infestations and mitigating the transmission risk of flea-borne pathogens to both animals and humans, in alignment with the One Health approach.

Despite the recognised impact of flea infestations on animal and human health, epidemiological data on their occurrence in dogs and cats in Portugal are very limited. The country has a temperate Mediterranean climate [20], predominantly characterised by mild winters and mild-hot summers, which hypothetically provides a favourable environment for flea populations all year round, particularly in places with high animal densities. Thus, the aim of the present study was to estimate the prevalence of flea infestation in dogs and cats in mainland Portugal, identify potential associated risk factors and carry out morphological and molecular characterisation of the flea specimens collected.

## Methods

### Study design, sampling and data collection

A cross-sectional study was conducted over a 1-year period, from March 2022 to February 2023. Sampling was carried out across rural and non-rural counties among the five NUTS II regions (Nomenclature of Units for Territorial Statistics II: North, Centre, Lisbon Metropolitan Area [LMA], Alentejo and Algarve) of mainland Portugal. Within each county, one veterinary clinic or hospital and one animal shelter, kennel or association were recruited for participation in the study.

Each participating sampling site received a sampling kit containing all necessary material (flea comb, animal data sheets, 2-ml tubes containing 70% ethanol, animal data sheets and shipping envelopes), along with detailed instructions, including a tutorial video accessible via QR code. Each month, three dogs and three cats were randomly selected per site and screened for flea infestation. Any dog or cat whose legal detainer consented to participate was considered eligible for the study. The collected samples and data sheets were sent by post to the Instituto de Higiene e Medicina Tropical (IHMT), Universidade NOVA de Lisboa, Portugal.

Epidemiological data for each sampled animal were recorded using structured data sheets, encompassing the following variables: species, sex, age, fur length, body condition, geographical origin, lifestyle, contact with other animals, sampling season, flea allergy dermatitis signs and history of antiparasitic treatment. Animals were classified as “treated” based on owner/legal detainer-reported use of an insecticide within the expected duration of efficacy and taking into consideration the stated active ingredient or commercial formulation and the time elapsed since its last application or administration.

### Flea collection and morphological identification

Each animal was combed 5 times using a fine-toothed flea comb moistened with 70% ethanol, focusing on the back, abdomen, hind limbs and inner limb surfaces, following guidelines from the World Association for the Advancement of Veterinary Parasitology (WAAVP) [21], with minor adaptions. Collected fleas were placed in 2-ml tubes containing 70% ethanol and sent to IHMT, where they were stored at room temperature until morphological identification.

All collected fleas were individually examined by stereomicroscopy and identified to the species level using dichotomous keys [13, 22–24]. Fleas of the same species collected from the same host were pooled in a single tube containing 70% ethanol and stored at room temperature for further analyses.

### DNA extraction and exoskeleton mounting of fleas

A subset of fleas was randomly selected for molecular analysis, with the aim to include three specimens per combination of flea species, host (dog or cat), NUTS II region and parish type (rural or non-rural, as defined by the Programa de Desenvolvimento Rural of the Portuguese Ministry of Agriculture and the Sea [PRODER] [25]), whenever available.

Total DNA was extracted while preserving flea exoskeletons [26] using the NZY Tissue gDNA Isolation kit (Nzytech, Lisbon, Portugal), with the following modifications. Each specimen was placed on a microscope slide and, under stereomicroscopic observation, a partial incision was made at the anterior dorsal region of the abdomen using a sterile disposable scalpel blade (No. 23). The specimen was then transferred to a 1.5-ml sterile tube containing 180 µl of NT1 buffer (NZYtech) and 25 µl of proteinase K (20 mg/ml; NZYtech) and incubated in a water bath at 56 ºC for 3 h with periodic agitation. After 90 min of incubation, an additional 16 µl of proteinase K (50 mg/ml; GRiSP, Porto, Portugal) was added. Once the digestion process was completed, the supernatant was transferred to a new 1.5-ml sterile tube, and DNA extraction was completed according to the manufacturer's instructions.

The retained exoskeleton was washed with 70% ethanol, dried on a paper towel and mounted on slides containing Hoyer’s medium for further morphological analysis.

### Amplification and sequencing of the mitochondrial cytochrome* c* oxidase II gene

Partial amplification of the mitochondrial cytochrome* c* oxidase subunit II (*cox2)* gene (770 bp) was performed by PCR using previously described primers: F-Leu (5’- TCTAATATGGCAGATTAGTGC-3’) and R-lys 5’- GAGACCAGTACTTGCTTTCAGTCATC-3’) [27].

PCR reactions were carried out in a final reaction volume of 25 µl (12.5 µl of NZYTaq II 2× Green Master Mix (NZYtech), 0.4 µM of each primer and 2 µl of template DNA). The thermal cycling conditions consisted of an initial denaturation at 95 °C for 12 min; followed by 37 cycles of denaturation at 94 °C for 45 s, annealing at 42 °C for 45 s, and extension at 68 °C for 2 min; with a final extension at 68 °C for 2 min. PCR products were visualised in a 1.5% agarose gel stained with GreenSafe Premium (NZYtech). Amplicons were sent to STAB VIDA (Caparica, Portugal), a commercial biotechnology company, for purification and Sanger sequencing, with the same primers as used in the DNA amplification.

### Sequence analysis and phylogenetics

Homologous sequences were identified using the BLASTn tool (https://blast.ncbi.nlm.nih.gov/Blast.cgi). Multiple sequence alignments were generated using the iterative G-INS-i refinement method implemented in MAFFT v7. The resulting alignment was processed with Gblocks [28] via SeaView v5.0.5 using default parameters, followed by manual editing using AliView v1.28 to maintain the correct open reading frame.

Phylogenetic analysis was conducted in IQ-TREE v1.6.12 using the maximum likelihood method, considering the best-fit evolutionary model selected according to the Bayesian Information Criterion. The reliability of the inferred tree was assessed by bootstrap resampling with 1000 replicates. The resulting phylogenetic trees were visualised and edited for display using iTOL v6 [29].

Representative sequences obtained in this study were submitted to the DNA Data Bank of Japan (DDBJ) and are available in the DDBJ/ENA/GenBank databases under the accession numbers (LC871646-LC871720). All sequences included in the phylogenetic analysis are listed in Additional file 1: Table S1.

### Statistical analysis

Data from animal record sheets and flea morphological identification records were entered into Microsoft Excel v365 (Microsoft Corp., Redmond, WA, USA) and subsequently imported into IBM SPSS (v25; IBM SPSS, Chicago, IL, USA), for statistical analysis.

Exploratory data analysis and descriptive statistics were performed to summarise the main variables of the dataset. Wilson’s method was used to calculate 95% confidence intervals (95% CIs) for prevalences. Associations between categorical variables (i.e. age group, body condition, cat/dog contact, fur length, indoor/outdoor animal contact, insecticide use, lifestyle, parish type, region, season, sex, species) were assessed using the Chi-square test (χ^2^) or the alternative Fisher’s exact test for 2 × 2 tables or Fisher-Freeman-Halton test for larger contingency tables. Adjusted standardised residuals (ASR) were calculated, with values exceeding ± 1.96 considered to be indicative of statistically significant contributions at α = 0.05.

The intensity range of flea infestation was determined by dividing the total number of fleas collected by the number of infested animals. The hypothesis that the presence of clinical signs compatible with FAD is associated with flea burden was tested using the Mann–Whitney U test, at α = 0.05. Age groups were defined according to reference categories previously established [30, 31].

Following the univariate analyses, a multivariate logistic regression model was developed to assess potential risk factors associated with flea infestation in dogs and cats. Variables with a *P* ≤ 0.200 in the univariate analysis were included in the multiple logistic regression model. A backward stepwise elimination procedure was applied, with a threshold of *P* ≤ 0.050 for retaining variables in the final model. For those variables the adjusted odds ratio (aOR) with 95% CI was determined. Model validity was assessed with the Hosmer–Leme (HL) goodness-of-fit test, the likelihood ratio test (*G*^2^) and the area under the receiver operating characteristic curve (AUC).

## Results

### Flea infestation prevalence and patterns

A total of 1052 dogs and 1039 cats from 94 counties (64 rural and 43 non-rural) were examined, with an overall flea infestation prevalence of 33.6% (353/1052) in dogs and 36.5% (379/1039) in cats (Tables 1, 2). No statistically significant difference was observed between dogs and cats in terms of infestation prevalence (χ^2^ = 2.05, *df* = 1, *P* = 0.153).

**Table 1:** Prevalence of flea infestation in dogs according to host characteristics, environment and insecticide use

Variable/categories	Examined, *n* (%)	Infested, *n* (%; 95% CI)	*P*-value/ASR
*Sex*	957		χ^2^ = 0.36, *df* = 1,* P* = 0.550
Female	469 (49.0)	151 (32.2; 28.1–36.6)	− 0.6
Male	488 (51.0)	166 (34.0; 30.0–38.3)	0.6
*Age group*	965		*P* = 0.015*^a^
Puppy	96 (23.3)	44 (45.8; 36.2–55.8)	2.8
Juvenile	149 (15.4)	51 (34.2; 27.1–42.2)	0.3
Young adult	2 (0.2)	2 (100; 34.2–100)	2.0
Mature adult	419 (43.4)	127 (30.3; 26.1–34.9)	− 1.6
Senior	225 (23.3)	68 (30.2; 24.6–36.5)	− 1.1
Geriatric	74 (7.7)	28 (37.8; 27.6–49.2)	0.9
*Fur length*	995		χ^2^ = 14.95, *df* = 2,* P* = 0.001*
Short	540 (54.3)	151 (28.0; 24.3–31.9)	− 3.8
Medium	346 (34.8)	133 (38.4; 33.5–43.7)	2.6
Long	109 (11.0)	46 (42.2; 33.4–51.6)	2.1
*Body condition*	1004		χ^2^ = 10.17, *df* = 4,* P* = 0.038*
Very thin	25 (2.5)	13 (52.0; 33.5–70.0)	2.0
Thin	132 (13.1)	53 (40.2; 32.2–48.7)	1.8
Ideal	644 (64.1)	211 (32.8; 29.3–36.5)	− 0.5
Overweight	182 (18.1)	53 (29.1; 23.0–36.1)	− 1.3
Obese	21 (2.1)	4 (19.0; 7.7–40.0)	− 1.4
*Region—NUTS II*	1044		χ^2^ = 69.74, *df* = 4,* P* < 0.0001*
North	317 (30.4)	143 (45.1; 39.7–50.6)	5.3
Center	172 (16.5)	67 (39.0; 32.0–46.4)	1.7
Lisbon Metropolitan Area	192 (18.4)	19 (9.9; 6.4–14.9)	− 7.6
Alentejo	149 (14.3)	49 (32.9; 25.9–40.8)	− 0.1
Algarve	214 (20.5)	70 (32.7; 26.8–39.3)	− 0.2
*Parish type*	1002		χ^2^ = 0.09, *df* = 1,* P* = 0.765
Rural	537 (53.6)	178 (33.1; 29.3–37.2)	0.3
Non-rural	465 (46.4)	150 (32.3; 28.2–36.6)	− 0.3
*Lifestyle*	1039		χ^2^ = 36.78, *df* = 4,* P* < 0.0001*
Sheltered	473 (45.5)	116 (24.5; 20.9–28.6)	− 5.4
Stray	15 (1.4)	5 (33.3; 15.2–58.3)	0.5
Domestic indoor	69 (6.6)	20 (29.0; 19.6–40.6)	− 0.8
Domestic indoor-outdoor	398 (38.3)	161 (40.5; 35.7–45.3)	4.0
Domestic outdoor	84 (8.1)	42 (50.0; 39.5–60.5)	3.4
*Indoor animal contact*	349		χ^2^ = 4.76, *df* = 1,* P* = 0.029*
No	104 (29.8)	49 (47.1; 37.8–56.6)	2.2
Yes	245 (70.2)	85 (34.7; 29.0–40.8)	− 2.2
*Outdoor animal contact*	374		χ^2^ = 7.86, *df* = 1,* P* = 0.005*
No	85 (22.7)	26 (30.6; 21.8–41.0)	− 2.8
Yes	289 (77.3)	138 (47.8; 42.1–53.5)	2.8
*Dog contact*	720		χ^2^ = 2.02, *df* = 1,* P* = 0.155
No	39 (5.4)	15 (38.5; 24.9–54.1)	1.4
Yes	681 (94.6)	190 (27.9; 24.7–31.4)	− 1.4
*Cat contact*	520		χ^2^ = 14.28, *df* = 1,* P* < 0.0001*
No	290 (55.8)	70 (24.1; 19.6–29.4)	− 3.8
Yes	230 (44.2)	91 (39.6; 33.5–46.0)	3.8
*Season*	1044		χ^2^ = 16.49, *df* = 4,* P* = 0.001*
Spring	370 (35.4)	107 (28.9; 24.5–33.7)	− 2.2
Summer	271 (26.0)	106 (39.1; 33.5–45.0)	2.4
Autumn	228 (21.8)	90 (39.5; 33.4–45.9)	2.3
Winter	175 (16.8)	44 (25.1; 19.3–32.1)	− 2.5
*Insecticide use*	715		χ^2^ = 23.7, *df* = 1,* P* < 0.0001*
No	64 (9.0)	32 (50.0; 38.1–61.9)	4.9
Yes	651 (91.0)	146 (22.4; 19.4–25.8)	− 4.9
Total	1052	353 (33.6; 30.8–36.5)	

*ASR* Adjusted standardised residuals, *CI* Confidence interval, *NUTS* Nomenclature of Units for Territorial Statistics

*Statistically significant at α = 0.05

^a^*P*-value derived from the Fisher-Freeman-Halton test

**Table 2:** Prevalence of flea infestation in cats according to host characteristics, environment, and insecticide use

Variable/categories	Examined, *n* (%)	Infested, *n* (%; 95% CI)	*P*-value/ASR
*Sex*	969		χ^2^ = 0.158, *df* = 1,* P* = 0.691
Female	486 (50.2)	177 (36.4; 32.2–40.8)	0.4
Male	483 (49.8)	170 (35.2; 31.1–39.6)	− 0.4
*Age group*	944		χ^2^ = 21.46, *df* = 5,* P* = 0.001*
Kitten	134 (14.2)	67 (50.0; 41.7–58.3)	3.6
Junior	333 (35.3)	132 (39.6; 34.5–45.0)	1.7
Adult	314 (33.3)	94 (29.9; 25.1–35.2)	− 2.8
Mature	120 (12.7)	35 (29.2; 21.8–37.8)	− 1.7
Senior	35 (3.7)	11 (31.4; 18.6–48.0)	− 0.6
Geriatric	8 (0.8)	2 (25.0; 7.1–59.1)	− 0.7
Fur length	953		*P* = 0.274^a^
Hairless	2 (0.2)	2 (100; 34.2–100)	1.8
Short	697 (73.1)	251 (36.0; 32.7–46.5)	− 1.0
Medium	188 (19.7)	74 (39.4; 32.7–46.5)	0.8
Long	66 (6.9)	25 (37.9; 21.8–43.8)	0.2
*Body condition*	985		χ^2^ = 11.74, *df* = 4,* P* = 0.019*
Very thin	14 (1.4)	7 (50.0; 26.8–73.2)	1.1
Thin	185 (18.8)	81 (43.8; 36.8–51.0)	2.3
Ideal	592 (60.1)	216 (36.5; 32.7–40.4)	0.1
Overweight	168 (17.1)	46 (27.4; 21.2–34.6)	− 2.7
Obese	26 (2.6)	8 (30.8; 16.5–50.0)	− 0.6
*Region—NUTS II*	1032		χ^2^ = 31.67, *df* = 4,* P* < 0.0001*
North	309 (29.9)	139 (45.0; 39.5–50.6)	3.6
Center	165 (16.0)	60 (36.4; 29.4–43.9)	− 0.1
Lisbon Metropolitan Area	185 (17.9)	37 (20.0; 14.9–26.3)	− 5.2
Alentejo	153 (14.8)	58 (37.9; 30.6–45.8)	0.4
Algarve	220 (21.3)	84 (38.2; 32.0–44.8)	0.5
*Parish type*	1000		χ^2^ = 0.75, *df* = 1,* P* = 0.387
Rural	472 (47.2)	176 (37.3; 33.0–41.7)	0.9
Non-rural	528 (52.8)	183 (34.7; 30.7–38.8)	− 0.9
*Lifestyle*	1025		χ^2^ = 98.77, *df* = 4,* P* < 0.0001*
Sheltered	364 (35.5)	100 (27.5; 23.1–32.3)	− 4.4
Stray	139 (13.6)	70 (50.4; 42.2–58.5)	3.7
Domestic indoor	216 (21.1)	47 (21.8; 16.8–27.7)	− 5.0
Domestic indoor-outdoor	241 (23.5)	104 (43.2; 37.1–49.5)	2.5
Domestic outdoor	65 (6.3)	51 (78.5; 67.0–86.7)	7.3
*Indoor animal contact*	313		χ^2^ = 19.77, *df* = 1,* P* < 0.0001*
No	85 (27.2)	49 (57.6; 47.0–67.6)	4.4
Yes	228 (72.8)	69 (30.3; 24.7–36.5)	− 4.4
*Outdoor animal contact*	297		χ^2^ = 18.71, *df* = 1,* P* < 0.0001*
No	83 (27.9)	20 (24.1; 16.2–34.3)	− 4.3
Yes	214 (72.1)	111 (51.9; 45.2–58.5)	4.3
*Dog contact*	506		χ^2^ = 0.26, *df* = 1,* P* = 0.609
No	289 (57.1)	113 (39.1; 33.7–44.8)	0.5
Yes	217 (42.9)	80 (36.9; 30.7–43.5)	− 0.5
*Cat contact*	752	*P* = 0.315	χ^2^ = 1.01, *df* = 1,* P* = 0.315
No	22 (2.9)	10 (45.5; 26.9–65.3)	1.0
Yes	730 (97.1)	256 (35.1; 30.7–43.5)	− 1.0
*Season*	1036		χ^2^ = 7.15, *df* = 3,* P* = 0.067
Spring	369 (35.6)	134 (36.3; 31.6–41.3)	− 0.1
Summer	284 (27.4)	120 (42.3; 36.6–48.1)	2.4
Autumn	221 (21.3)	74 (7.1; 27.6–39.9)	− 1.0
Winter	162 (15.6)	40 (4.8; 18.7–31.9)	− 1.6
*Insecticide use*	427	*P* < 0.001	χ^2^ = 21.78, *df* = 1,* P* < 0.0001*
No	84 (14.7)	39 (46.4; 36.2–57.0)	4.7
Yes	487 (85.3)	105 (18.4; 18.1–25.4)	− 4.7
Total	1040	379 (36.5; 33.6–39.5)	

^a^*ASR* Adjusted standardised residuals, *CI* Confidence interval, *NUTS* Nomenclature of Units for Territorial Statistics

*Statistically significant at α = 0.05

^a^*P*-value derived from the Fisher-Freeman-Halton test

The prevalence of flea infestation did not differ significantly between sexes in either species (dogs: χ^2^ = 0.36, *df* = 1, *P* = 0.550; cats: χ^2^ = 0.16, *df* = 1, *P* = 0.691). In dogs, 32.2% of females (151/469) and 34.0% of males (166/488) were infested. In cats, infestation was recorded in 36.4% of females (177/486) and 35.2% of males (170/483).

A significant association was observed between flea infestation and age group in both dogs (*P* = 0.015) and cats (χ^2^ = 21.46, *df* = 5, *P* = 0.001). Puppies (age < 6 months; 45.8%, 44/96, ASR = 2.8) and kittens (age ≤ 6 months; 50.0%, 67/134, ASR = 3.6) exhibited the highest prevalence of infestation.

In dogs, fur length was significantly associated with infestation (χ^2^ = 14.95, *df* = 2, *P* = 0.001), with short-haired individuals presenting the lowest prevalence of infestation (28.0%, 151/540, ASR = − 3.8).

Lower body condition scores were significantly associated with higher flea infestation prevalence in both dogs (42.0%, 66/157, χ^2^ = 7.98, *df* = 2, *P* = 0.019, ASR = 2.5) and cats (44.2%, 88/199, χ^2^ = 11.41, *df* = 2, *P* = 0.003, ASR = 2.6).

Geographical origin also influenced flea infestation prevalence, with the highest values recorded in the North (dogs: 45.1%, 143/317, χ^2^ = 69.74, *df* = 4, *P* < 0.0001, ASR = 5.3; cats: 45.0%, 139/309, χ^2^ = 31.67, *df* = 4, *P* < 0.0001, ASR = 3.6) and the lowest recorded in the LMA (dogs: 9.9%, 19/192, ASR = − 7.6; cats: 20.0%, 37/185, ASR = − 5.2). In contrast, no significant differences were found regarding parish type (dogs: χ^2^ = 0.09, *df* = 1, *P* = 0.765; cats: χ^2^ = 0.75, *df* = 1, *P* = 0.387).

Lifestyle was significantly associated with flea infestation (dogs: χ^2^ = 36.78, *df* = 4, *P* < 0.0001; cats: χ^2^ = 98.77, *df* = 4, *P* < 0.0001). Outdoor domestic dogs and cats exhibited the highest prevalence (cats: 78.5%, 51/65, ASR = 7.3; dogs: 50.0%, 42/84, ASR = 3.4), whereas sheltered individuals had the lowest (cats: 27.5%, 100/364, ASR = - 4.4; dogs: 24.5%, 116/473, ASR = - 5.4). Additionally, dogs in contact with outdoor animals had a significantly higher infestation prevalence compared to those without such contact (χ^2^ = 7.86, *df* = 1, *P* = 0.005; 47.8%, 138/289, ASR = 2.8 vs. 30.6%, 26/85, ASR = − 2.8), and those in contact with cats were more frequently infested (χ^2^ = 14.282, *df* = 1, *P* < 0.0001; 39.6%, 91/230, ASR = 3.8 vs. 24.1%, 70/290, ASR = - 3.8).

In contrast to cats, seasonality significantly influenced flea infestation in dogs (χ^2^ = 16.49, *df* = 3, *P* = 0.001), with the highest prevalence observed in summer (39.1%, 106/271, ASR = 2.4) and autumn (39.5%, 90/228, ASR = 2.3), while the lowest infestation prevalence was in winter (25.1%, 44/175, ASR = − 2.5).

### Flea species

**Fig. 1:**
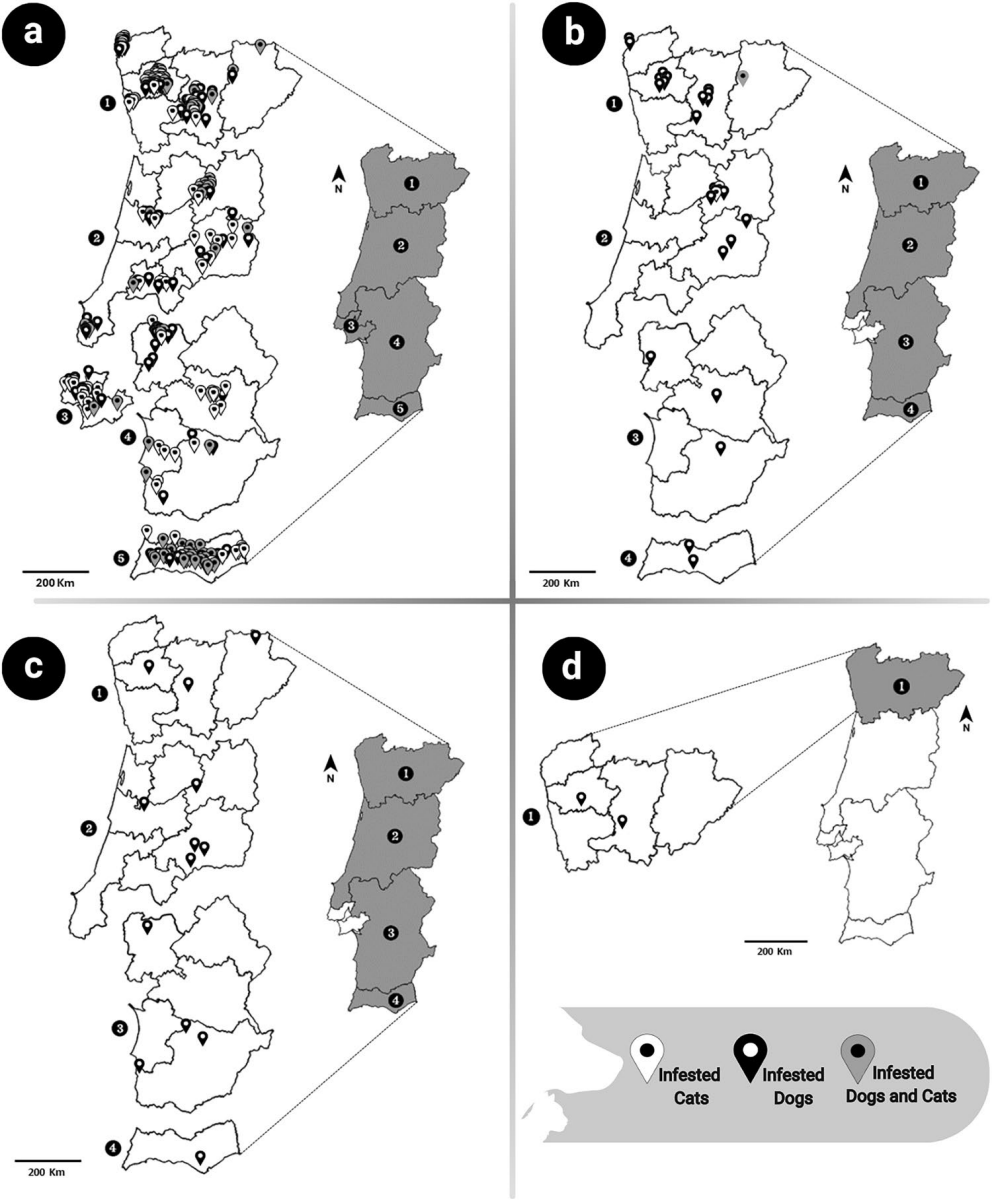
Geographic distribution of flea species detected in infested hosts, by regions classified according to the Nomenclature of Units for Territorial Statistics level II (NUTS II). **A*** Ctenocephalides felis*;** B*** Ctenocephalides canis*;** C*** Pulex irritans*;** D*** Archaeopsylla erinacei maura*.

A total of 1513 flea specimens were collected from dogs (51.8%, 784/1513) and cats (48.2%, 729/1513) (Fig. 1). *Ctenocephalides felis* was the most frequently identified flea species, accounting for 85.7% (672/784) of fleas in dogs and 98.8% (720/729) in cats (Table 3). Other species identified included *C. canis* (7.8%, 61/784 in dogs; 1.2%, 9/729 in cats), *P. irritans* (5.5%, 43/784, exclusively in dogs) and *A. erinacei maura* (1.0%, 8/784, exclusively in dogs).

**Table 3:** Prevalence and mean intensity of flea infestation by flea species in dogs and cats

Infestation type/flea species	Dog		Cat	
	Prevalence, *n* (%; 95% CI)	Intensity^a^ (range)	Prevalence, *n* (%; 95% CI)	Intensity^a^ (range)
*Monoinfestation*	342 (96.9; 94.5–98.3)	2.1 (1–17)	379 (100.0; 99.0–100.0)	1.9 (1–33)
*Ctenocephalides felis*	302 (85.6; 81.5–88.8)	2.1 (1–17)	372 (98.2; 96.2–99.2)	1.9 (1–33)
*Ctenocephalides canis*	29 (8.2; 5.8–11.6)	1.9 (1–7)	7 (1.8; 0.9–3.7)	1.3 (1–3)
*Pulex irritans*	8 (2.3; 1.2–4.4)	3.4 (1–7)	0	na
*Archaeopsylla erinacei maura*	2 (0.6; 0.2–2.0)	3.5 (1–6)	0	na
*Co-infestation*	12 (3.4; 2.0–5.9)	4.6 (2–11)	0	na
*C. felis* + *P. irritans*	6 (1.7; 0.8–3.7)	4.7 (4–6)	0	na
*C. canis* + *C. felis*	4 (1.1; 0.4–2.9)	3.8 (2–7)	0	na
*A. erinacei maura* + *C. felis*	1 (0.3; 0.1–1.5)	2.0	0	na
*C. canis* + *C. felis* + *Pulex irritans*	1 (0.3; 0.1–1.5)	11.0	0	na
Total	353	2.2 (1–17)	379	1.9 (1–33)

^a^The mean number of fleas per infested animal

*CI* Confidence interval, *na* not applicable

Mono-infestations were predominant, with *C. felis* being the only flea species in 85.6% (302/353) of infested dogs and 98.2% (372/373) of infested cats. Co-infestations were observed only in dogs (3.4%, 12/353), most frequently involving *C. felis* and *P. irritans* (50.0%, 6/12), followed by *C. canis* and *C. felis* (33.3%, 4/12), *A. erinacei maura* and *C. felis* (8.3%, 1/12) and *C. canis*, *C. felis* and *P. irritans* (8.3%, 1/12).

Flea intensity ranged from 1 to 17 (mean 2.2) fleas per dog and from 1 to 33 (mean 1.9) fleas per cat.

### Molecular characterisation and phylogenetics

**Fig. 2:**
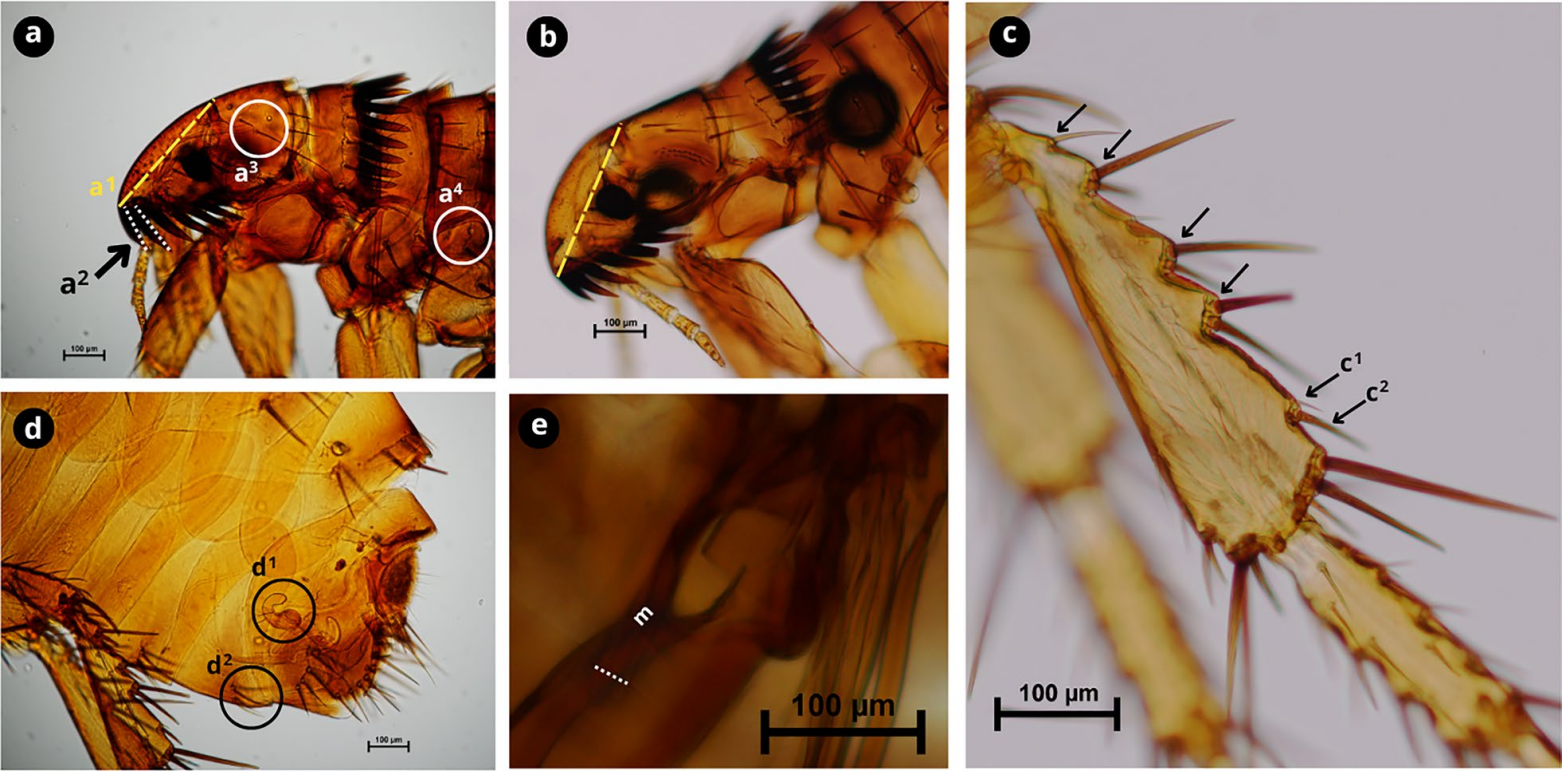
Morphological characteristics of *Ctenocephalides felis*.** a** Female cephalic capsule with a noticeable acute angle and not very convex front anteriorly (a^1^); first spine of the genal comb is approximately the same length as the second (a^2^); occiput area has two setae (a^3^); lateral metatorax area with one or two setae (a^4^).** b** Male front more convex anteriorly compared to the female.** c** Hind tibia with five groups of setae bearing notches on the dorsoposterior margin (black arrows) with vestigial spiniform setae c^1^ and developed seta c^2^.** d** Female genitalia; spermatheca (d^1^); sternite VII with two setae, one posterior and one anterior (d^2^).** e** Male genitalia, with the manubrium (m) not expanded apically with a constricted apex (white dashed line). Scale bars as shown in the Figure.

**Fig. 3:**
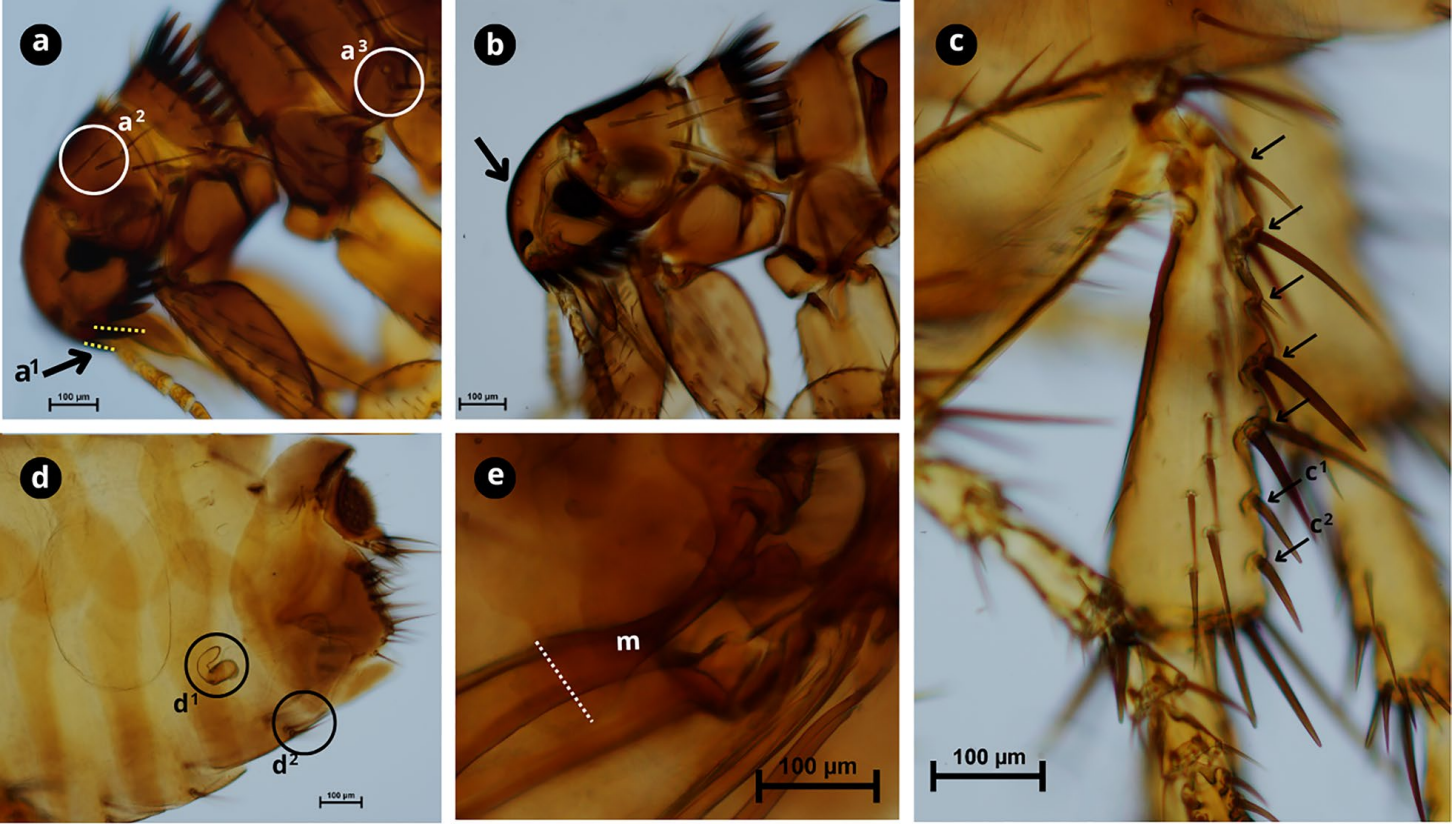
Morphological characteristics of *Ctenocephalides canis*.** a** First spine of the genal comb is half the length of the second (a^1^); occiput area has three setae (a^2^); lateral metatorax area with three setae (a^3^).** b** Cephalic capsule with a convex front anteriorly.** c** Hind tibia with six to seven groups of setae bearing notches on the dorsoposterior margin (black arrows) with developed spiniform setae c^1^ and c^2^.** d** Female genitalia; spermatheca (d^1^); sternite VII with two setae on the same level (d^2^).** e** Male genitalia, with manubrium (m) expanded apically with a dilated apex (white dashed line). Scale bars as shown in the Figure.

**Fig. 4:**
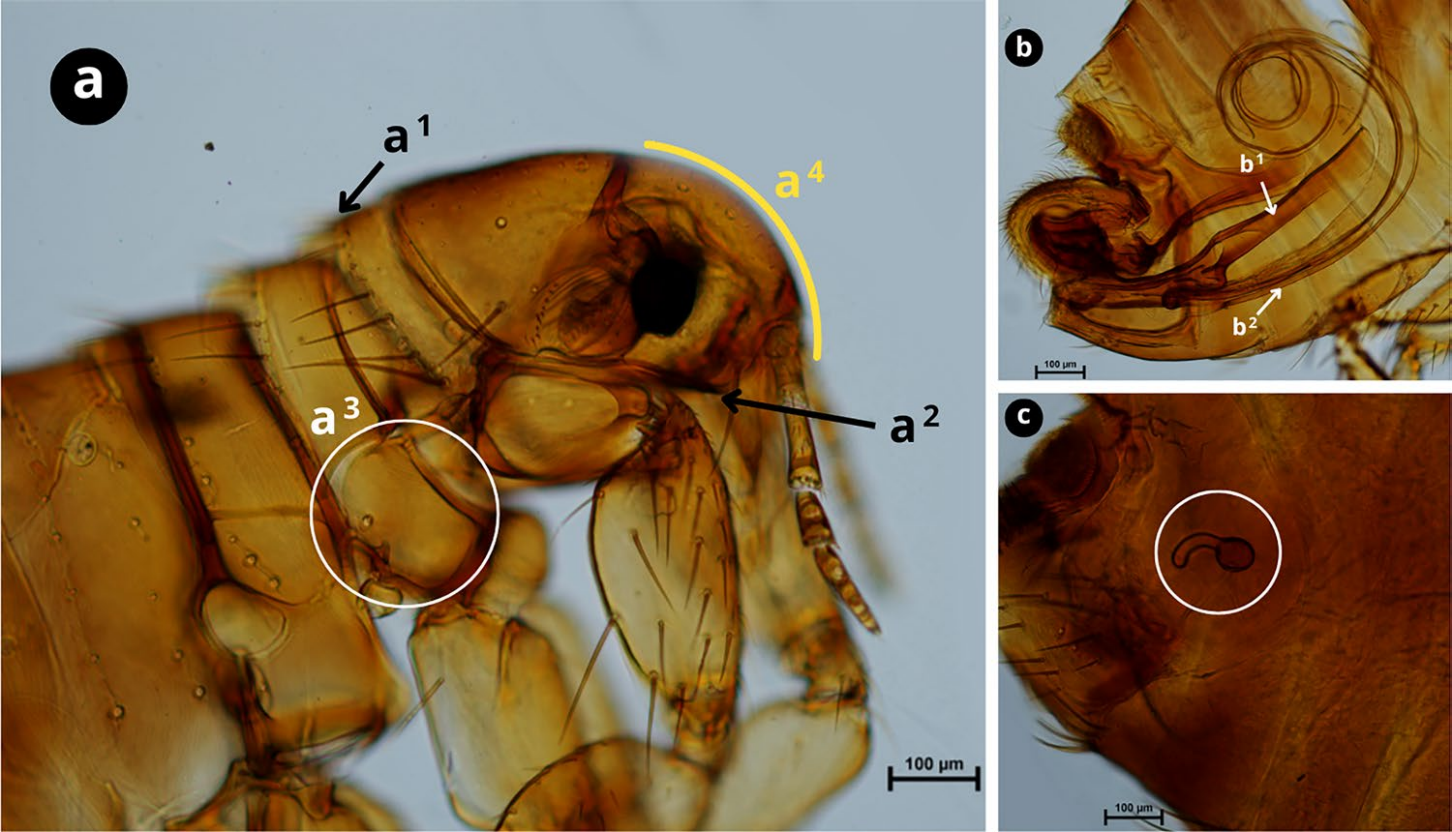
Morphological characteristics of* Pulex irritans*.** a** Pronotal and genal combs absent (a^1^ and a^2^); pleural rod of mesothorax absent (a^3^); rounded front (a^4^).** b** Male genitalia, phallosome (tubular and central) (b^1^) and accessory filaments (ventral) (b^2^).** c** Female genitalia, spermatheca. Scale bars as shown in the Figure.

**Fig. 5:**
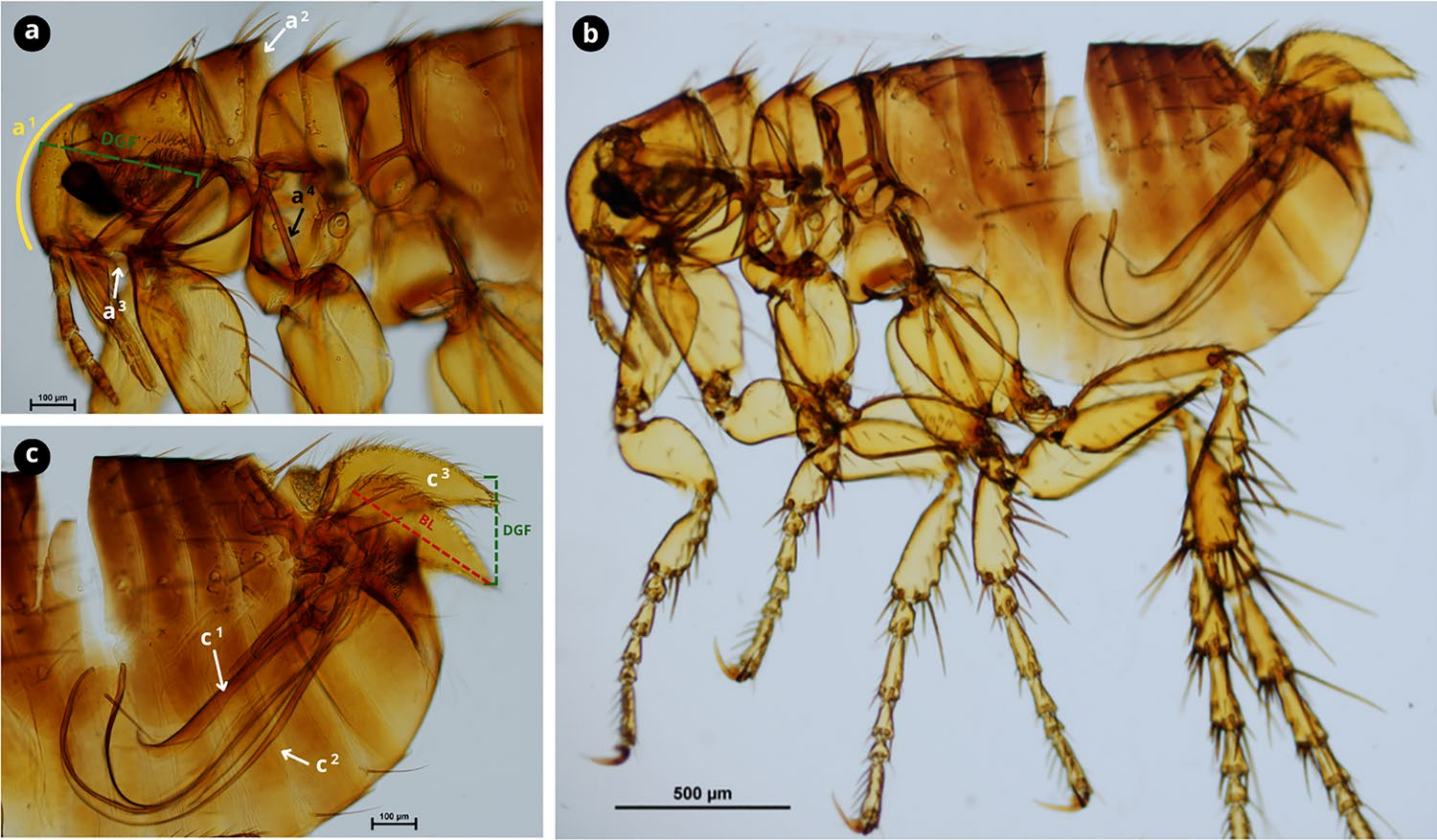
Morphological characteristics* Archaeopsylla erinacei maura* (male).** a** Pronotal comb absent or vestigial, with one or three combs on each side (a^1^); vestigial genal comb with one or three spines (a^2^); pleural rod of mesothorax present (a^3^); cephalic capsule with rounded front (a^4^); distance from base of spine at tip of genal process to front (DGF).** b** Whole body.** c** Phallosome (tubular and central) (c^1^) and accessory filaments (ventral) (c^2^); basimere (c^3^); basimere length (higher in the subspecies* A. erinacei maura compared* to* A. erinacei erinacei*) (BL), basimere length equal to DGF. Scale bars as shown in the Figure.

A total of 75 *cox2* sequences were obtained (100% amplification success) from flea specimens morphologically identified as *C. felis* (72.0%, 54/75), *C. canis* (13.3%, 10/75), *P. irritans* (13.3%, 10/75) and *A. erinacei maura* (1.3%, 1/75) (Figs. 2, 3, 4, 5).

**Fig. 6:**
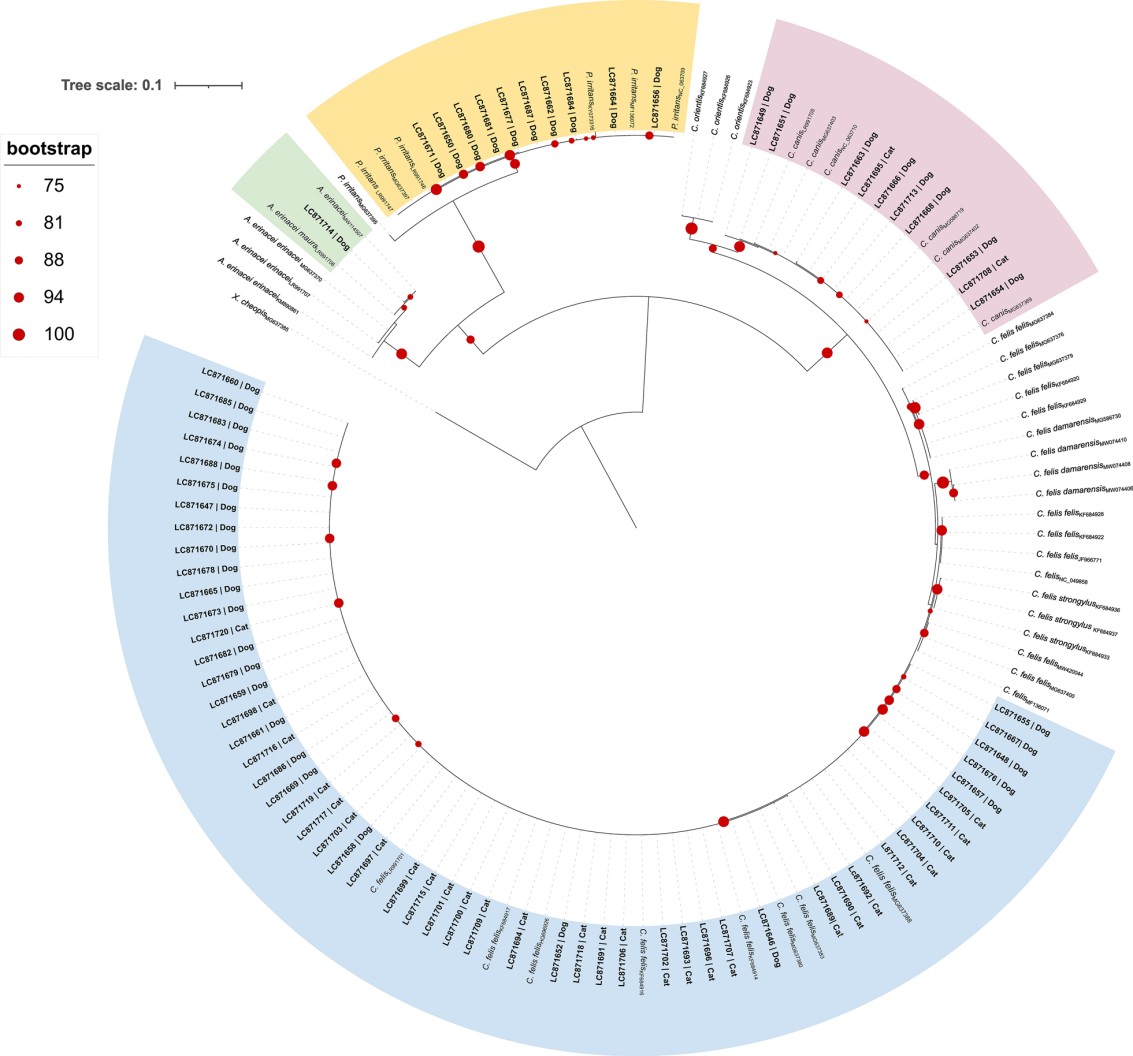
Maximum likelihood phylogenetic tree inferred from cytochrome oxidase subunit II (*cox2*) sequences of various flea species and subspecies. Tree reconstruction was performed in IQ-TREE using the K3Pu+F+G4 substitution model, selected as the best-fitting model based on the Bayesian Information Criterion. Node support was assessed using 1000 bootstrap replicates, and values ≥75% are shown at the corresponding nodes. The tree was rooted using* Xenopsylla cheopis* sequence (outgroup). Reference sequences are labelled with species name and GenBank accession number. Sequences obtained in this study are shown in bold and include specimen identifier and GenBank accession number (LC871646-LC871720). Branch lengths are scaled to the number of substitutions per site. Coloured sectors denote the phylogenetic clusters in which the sequences obtained in this study segregated

Phylogenetic analysis indicated that *C. felis* sequences shared a common ancestor. Nonetheless, *C. felis felis* showed a paraphyletic origin, with sequences obtained from morphologically identified *C. felis* specimens collected from dogs and cats segregating into a monophyletic cluster with low genetic divergence, composed exclusively of reference sequences of *C. felis felis* from dogs and cats in Australia, Hungary, Israel, Italy and Spain (Fig. 6).

*Ctenocephalides canis* showed a monophyletic origin, with the obtained sequences of *C. canis* from dogs and cats clustering together with reference sequences of *C. canis* collected from dogs and cats in China, the Czech Republic, Hungary, Iran and Turkey.

The obtained sequences of *P. irritans* from dogs segregated into a monophyletic cluster composed exclusively of sequences of *P. irritans* collected from humans and animals, Argentina, China, Croatia, Madagascar and Spain.

Similarly, the obtained sequence of *A. erinacei maura* collected from a dog formed a robust monophyletic cluster with sequences of *A. erinacei maura* from hedgehogs in Spain and Portugal.

### Flea allergy dermatitis in infested animals

Flea allergy dermatitis was significantly associated with flea infestation in both dogs and cats (dogs: χ^2^ = 71.81, *df* = 1, *P* < 0.0001; cats: χ^2^ = 16.02, *df* = 1, *P* < 0.0001. Infested animals had a higher prevalence of FAD compared to non-infested individuals (dogs: 16.1%, 53/330, ASR = 8.5 vs. 1.8%, 12/653, ASR = − 8.5); cats: 5.4%, 20/367, ASR = 4.0 vs. 1.1%, 7/616, ASR = - 4.0). Host species was also significantly associated with FAD occurrence (χ^2^ = 16.47, *df* = 1, *P* < 0.0001), with dogs showing a higher prevalence of FAD (6.6%, 65/983, ASR = 4.1) compared to cats (2.7%, 27/983; ASR = − 4.1). In addition, flea burden was significantly higher in animals presenting clinical signs compatible with FAD (dogs: *U* = 15098.00, *Z* = − 7.95,* P* < 0.0001; cats: *U* = 8168.00, *Z* = − 3.79, *P* < 0.0001).

### Effect of insecticide use

The use of insecticides was significantly associated with lower flea infestation in both dogs and cats (dogs: χ^2^ = 23.69, *df* = 1, *P* =  < 0.001; cats: χ^2^ = 21.78, *df* = 1, *P* =  < 0.001).

Infestation prevalence was lower in treated animals (dogs: 22.4%, 146/651, ASR = − 4.9; cats: 21.7%, 106/488, ASR = = − 4.7) compared to untreated individuals (dogs: 50.0%, 32/64, ASR = 4.9; cats: 45.8%, 38/83, ASR = = 4.7), indicating a protective effect of insecticide application. Additionally, a significant association was observed between insecticidal use and flea infestation in both species (*P* < 0.001). Animals treated with fipronil showed the highest ASR (dogs = 6.0; cats = 8.7), compared to all other treatment groups (Additional file 2: Table S1, Table S2).

### Multivariable logistic regression analysis of risk factors for flea infestation

**Fig. 7:**
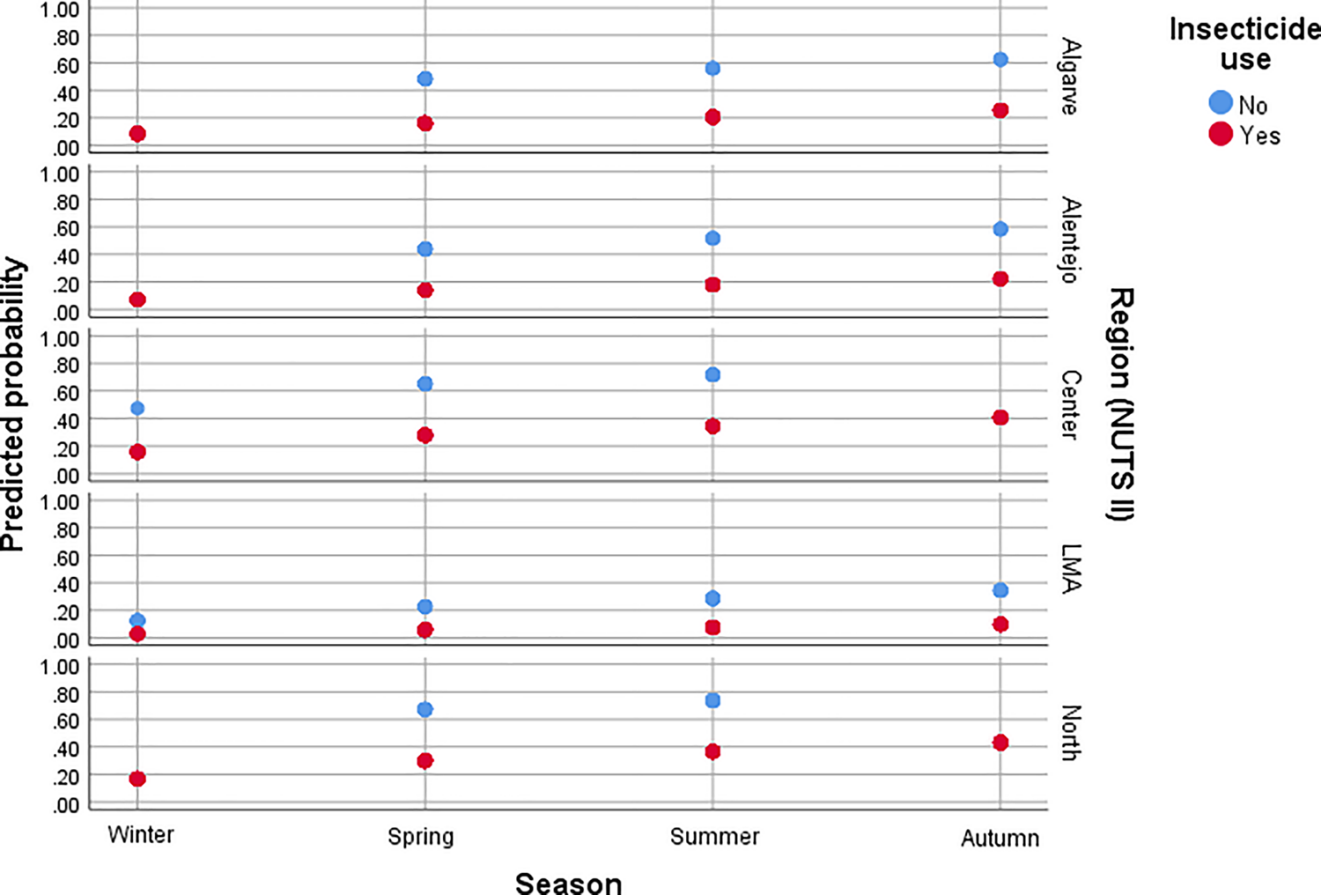
Predicted probability of flea infestation in dogs according to NUTS II region, season, and insecticide use.

**Fig. 8:**
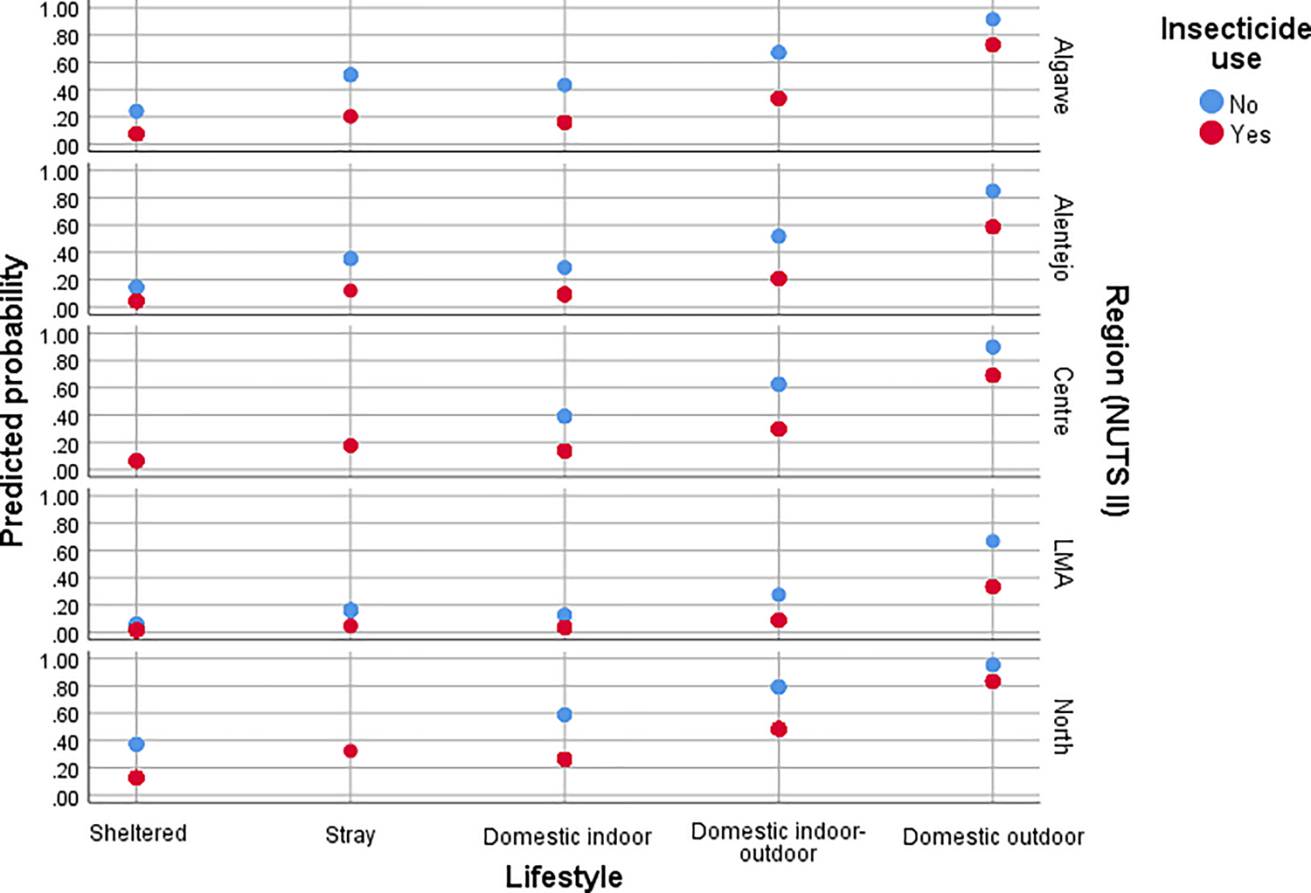
Predicted probability of flea infestation in cats according to NUTS II region, lifestyle, and insecticide use.

The multivariate logistic regression analysis identified NUTS II/seasonality/insecticide use and NUTS II/lifestyle/insecticide use as significant predictors of flea infestation in dogs (*G*^2^ = 85.22, *df* = 8, *P* < 0.0001) and cats (*G*^2^ = 136.12, *df* = 9, *P* < 0.0001), respectively (Additional file 2: Table S3, Table S4; Figs. 7, 8).

Regarding infestation according to NUTS II regions in mainland Portugal, compared to the North region, the odds of flea infestation were significantly lower in LMA (χ^2^_Wald_ = 31.85, *df* = 1, *P* < 0.0001, aOR = 0.14, 95% CI 0.21–0.69), Alentejo (χ^2^_Wald_ = 10.35, *df* = 1, *P* = 0.001, aOR = 0.38, 95% CI 0.21–0.69) and Algarve (χ^2^_Wald_ = 8.81, *df* = 1, *P* = 0.003, aOR = 0.45, 95% CI 0.27–0.76) for dogs, and in LMA (χ^2^_Wald_ = 24.73, *df* = 1, *P* < 0.0001, aOR = 0.10, 95% CI 0.41–0.25), Alentejo (χ^2^_Wald_ = 10.08, *df* = 1, *P* = 0.001, aOR = 0.28, 95% CI 0.13–0.62), and Centro (χ^2^_Wald_ = 7.08, *df* = 1, *P* = 0.008, aOR = 0.45, 95% CI 0.25–0.81) for cats.

The odds of flea infestation in dogs were significantly higher in spring (χ^2^_Wald_ = 4.60, *df* = 1, *P* = 0.032, aOR = 2.08, 95% CI 1.07–4.06), summer (χ^2^_Wald_ = 8.89, *df* = 1, *P* = 0.003, aOR = 2.83, 95% CI 1.43–5.61) and autumn (χ^2^_Wald_ = 13.85, *df* = 1, *P* < 0.0001, aOR = 3.72, 95% CI 1.86–7.43), compared to winter.

In cats, lifestyle was a significant predictor of flea infestation (χ^2^_Wald_ = 53.93, *df* = 4, *P*  < 0.0001), with domestic cats having outdoor access exhibiting the highest odds of infestation.

Flea infestation was associated with the absence of insecticide use in both dogs and cats. Compared to treated individuals, untreated dogs had nearly a fivefold higher odds of infestation (χ^2^_Wald_ = 27.57, *df* = 1, *P* < 0.0001, aOR = 4.87, 95% CI 2.70–8.79), while untreated cats had approximately a fourfold higher odds of infestation (χ^2^_Wald_ = 17.88, *df* = 1, *P* < 0.0001, aOR = 4.02, 95% CI 2.11–8.67).

The adjusted models demonstrated a good fit to the data (dogs: χ^2^_HL_ = 7.96, *df* = 8, *P* = 0.438; cats: χ^2^_HL_ = 9.72, *df* = 7, *P* = 0.205) and exhibited adequate discriminative performance (dogs: AUC = 0.71, *P* < 0.0001; cats: AUC = 0.77, *P* < 0.0001).

## Discussion

To the best of our knowledge, this study represents the most extensive epidemiological investigation of flea infestations in dogs and cats conducted to date in mainland Portugal. The results provide new insights into prevalence, associated risk factors and molecular diversity of flea infestations.

In this study, a total of 1052 dogs and 1039 cats were examined, with an overall flea infestation prevalence of 36.5% in cats and 33.6% in dogs. These values are within the range of those reported in previous studies conducted in European countries [2, 9, 15–18, 32]. Lower prevalence has been observed in the UK (21.1–28.1% in cats and 6.8–14.4% in dogs [4, 9]), Hungary (22.9% in cats; 14.1% in dogs [2]), Germany (14.3% in cats and 5.1% in dogs [32]) and Italy (17.9% in dogs [16]). Conversely, higher prevalence has been reported in Spain (41.8% in dogs [15]), Cyprus (39.1% in dogs [18]) and Greece (40.3% in dogs; 97.4% in cats [17]). These geographic differences likely result from a combination of climatic, environmental and management factors. Temperature and humidity are critical determinants of flea population dynamics, influencing survival, development and reproduction [7].

Four flea species were identified in this study, with *C. felis* predominating in both hosts, accounting for 98.8% (720/729) of fleas collected in cats and 85.7% (672/784) of fleas collected in dogs. These results are consistent with those of previous studies worldwide [9, 15, 16, 18, 19, 32], confirming *C. felis* as the most widespread and clinically important flea species in companion animals [12]. The broad host range and wide ecological plasticity of this flea species contribute to its dominance [7]. Moreover, *C. felis* is known to be a competent vector of several zoonotic pathogens, including *B. henselae*, the causative agent of cat scratch disease, and *R. felis*, responsible for flea-borne spotted fever [33]. It also serves as an intermediate host for *D. caninum* [6], underscoring the importance of flea control for both animals and public health.

*Ctenocephalides canis* was the second most frequently identified species in both the dogs and cats examined in this study, which is again consistent with results reported in previous studies [9, 34]. Its lower prevalence compared to *C. felis* may be due to a narrower environmental tolerance, with *C. canis* favouring cooler and wetter habitats [34]. Additionally, *C. canis* has been linked to contact with wild canids such as foxes, which may contribute to its higher prevalence in rural settings [2]. The ecological dominance of *C. felis* may also play a role, as interspecific competition is thought to limit the establishment and spread of other flea species in companion animals [15].

*Pulex irritans* was exclusively found in dogs and ranked as the third most common flea species identified in this study. Although traditionally associated with humans, *P. irritans* has also been reported in several domestic and wild animals and is considered to be an emerging flea species of veterinary relevance [2, 15]. Importantly, *P. irritans* has been implicated in the transmission of several zoonotic pathogens, including *Yersinia pestis*, the etiological agent of plague [35], further reinforcing its epidemiological significance [36].

*Archaeopsylla erinacei maura* was identified in three dogs, in line with previous reports describing its occasional occurrence in companion animals [9, 14, 32]. This flea species is primarily associated with hedgehogs [24], and its detection in dogs likely reflects incidental contact in rural or peri-urban environments where interactions between domestic animals and synanthropic wildlife may occur. These findings support the hypothesis that companion animals may act as bridging hosts for flea species typically associated with wildlife [37].

Most infestations were mono-infestations, consistent with prior reports [2], suggesting that *C. felis* can establish and maintain infestations without interspecific competition [15]. Co-infestations occurred exclusively in dogs (3.4%), and most frequently involved *C. felis* with either *P. irritans* (50.0%) or *C. canis* (33.3%). The absence of co-infestations in cats may reflect the high dominance of *C. felis* in felines, potentially indicating interspecific exclusion or reduced exposure to other flea species [14].

Molecular analysis played a crucial role in confirming flea species identity, particularly within the *Ctenocephalides* genus, where morphological differentiation is often challenging [13]. In this study, 75 morphologically identified fleas representing all detected species were randomly selected for molecular characterisation using the mitochondrial *cox2* gene. Amplification and sequencing were successful in all specimens, underscoring the reliability of the DNA extraction method and the sensitivity of the PCR protocol used. Both *cox1* and *cox2* genes are commonly used in molecular studies of fleas, offering comparable phylogenetic resolution at the species and subspecies levels [38, 39]. In this study, *cox2* was selected due to its consistently higher amplification success in previous studies, whereas *cox1* has been associated with occasional amplification failure and the generation of non-specific PCR products [38].

Phylogenetic analysis confirmed the morphological identifications, with the resulting tree showing distinct and well-supported monophyletic clusters for each flea species.

All specimens selected for molecular characterisation and morphologically identified as *C. felis* were confirmed to belong to the subspecies *C. felis felis*, segregating in a monophyletic cluster together with reference sequences of *C. felis felis* from Australia, Israel, Hungary, Italy and Spain. This cluster includes sequences from specimens previously classified as *C. felis felis* of the ‘Temperate’ lineage [13], which is globally widespread and likely disseminated through human-mediated movement of companion animals. Furthermore, the phylogenetic analysis suggests very low genetic diversity within the *C. felis felis* population infesting companion animals in Portugal, potentially reflecting the predominance of a single, well-adapted lineage.

Sequences of *C. canis* formed a monophyletic cluster with reference sequences from diverse geographic regions. No genetic differentiation was observed between specimens collected from dogs and cats, suggesting that although this species is primarily associated with canids, it is also adapted to infesting felines. As observed for *C. felis felis*, genetic diversity within *C. canis* was low, consistent with previous studies [39].

The *P. irritans* sequences obtained from the specimens analysed in this study clustered with those from specimens collected from humans and animals in Spain, Croatia, Madagascar, China and Argentina, supporting its broad host range. In contrast to previous studies [36, 39], no divergent lineages were identified. Lastly, the presence of *A. erinacei maura* in dogs was confirmed by phylogenetic analysis, which is in agreement with previous records [24], suggesting incidental cross-species transmission in synanthropic environments where domestic animals and wildlife interact [37].

Flea allergy dermatitis is one of the most common causes of skin disease in both dogs and cats in flea-endemic regions [40]. In the present study, the presence of clinical signs compatible with FAD was significantly associated with flea infestation, and more frequently observed in dogs than cats. These findings differ from those reported in the UK where cats exhibited a greater frequency of clinical signs compatible with FAD (8.0% vs. 3.3%) [4]​. While both dogs and cats can develop hypersensitivity to flea saliva allergens, experimental evidence suggests that cats typically develop skin lesions only under conditions of continuous exposure [41], whereas intermittent exposure is sufficient to trigger FAD in dogs [42]. Furthermore, the higher flea infestation intensity observed in dogs may result in increased antigenic stimulation, which could exacerbate hypersensitivity reactions and contribute to the higher prevalence of clinical FAD in dogs. Therefore, these results reinforce the importance of effective flea control programmes, not only to prevent parasitism but also to mitigate flea-associated dermatitis.

The univariate analysis identified several variables as being potentially associated with flea infestation in dogs and cats. However, in the multivariate logistic regression models, geographical region/lifestyle/insecticide use and geographical region/season/insecticide use were retained as significant predictors of flea infestation in cats and dogs, respectively. These findings highlight the importance of multivariate approaches in epidemiological studies, as they account for confounding factors and enable a more robust identification of risk factors [43].

Geographic variation in infestation prevalence was evident in the present study, with significantly lower odds of flea infestation in central and southern Portugal compared to the north. This pattern likely reflects climatic gradients, as the northern region experiences higher precipitation and relative humidity [44], which create more favourable microclimatic conditions for flea development [3]. These findings align with those of previous studies [3, 5] demonstrating regional differences in flea prevalence, linked to environmental and management factors.

Seasonality was a significant predictor of flea infestation in dogs, with markedly lower prevalence during the winter. Environmental temperature has a strong influence on flea life-cycles. Colder temperatures slow or inhibit the development of immature stages, reducing adult emergence [45]. Additionally, reduced outdoor activity and host contact in winter may limit flea transmission [5]. Interestingly, this seasonal pattern was not observed in cats, whose infestation prevalence remained relatively stable throughout the year. This likely reflects the buffering effect of indoor environments, which provide stable conditions that support flea development regardless of outdoor climate [3].

Lifestyle was also an important predictor of flea infestation in cats. Domestic cats with outdoor access were significantly more likely to be infested, underscoring the role of environmental exposure and potential contact with infested domestic or wild animals in increasing the risk of flea infestation [2]. Interestingly, stray and shelter cats showed lower infestation prevalence, which may be explained by the periodic administration of ectoparasiticides by caregivers in shelters and associations as well as by antiparasitic interventions commonly performed during Catch-Neuter-Return campaigns as part of routine veterinary protocols.

The use of insecticides was strongly associated with reduced odds of flea infestation in both dogs and cats, reinforcing the importance of regular parasite control. This finding is in line with previous research suggesting that insecticide use reduces flea infestation in companion animals [3]. Nonetheless, infestations were still observed in treated animals, pointing to potential issues such as suboptimal efficacy, poor compliance (e.g. incomplete/incorrect application, post-treatment shampooing or water immersion), or reinfestation from the environment or untreated hosts [3].

The owner-reported use of formulations containing permethrin in cats raises safety concerns as this compound is highly toxic to felines. A similar situation was reported in a previous study conducted in Portugal on pet owner deworming practices [46], likely reflecting confusion with dog-only products that share similar commercial names and packaging. These observations underscore the need for clearer labelling, precise veterinary guidance and owner education on correct product use to avoid inadvertent toxic exposures.

Interestingly, among the treated animals, those receiving fipronil exhibited the highest prevalence of infestation. Although fipronil has demonstrated high efficacy under controlled conditions, reduced performance in field settings has been increasingly suggested [3, 7]. While resistance to pyrethroids is well documented, evidence for confirmed resistance to fipronil remains limited [47]. Nevertheless, sporadic treatment failures may indicate emerging resistance or reflect other factors, such as incorrect application, suboptimal dosing or reinfestation from infested animals/environments [3]. These findings highlight the need for further investigation into the efficacy of commonly used insecticides under field conditions and reinforce the importance of ongoing surveillance for potential resistance development [21].

## Conclusions

The findings of this nationwide study highlights that flea infestations remain highly prevalent in both dogs and cats across mainland Portugal. *Ctenocephalides felis* was the predominant species in both hosts, with molecular data supporting its identification as *C. felis felis*. The absence of insecticide use was identified as the strongest predictor of flea infestation, and the association of infestation with clinical signs compatible with FAD underscores the clinical relevance of these ectoparasites. Although less common, other flea species were also detected, reinforcing the importance for ongoing entomological surveillance. These findings provide a robust epidemiological basis for targeted, evidence-based flea control strategies aimed at improving animal health and preventing associated complications. Continued efforts in veterinary education, owner compliance and monitoring of insecticide efficacy will be essential to reduce infestation pressure.

## Supplementary Information

**Additional file 1.**

**Additional file 2.**

## Data Availability

The datasets generated and analysed during the current study are not publicly available due to confidentiality commitment with the participants, as stated in the consent declaration, but are available from the corresponding author on reasonable request.

## Abbreviations

aOR: Adjusted odds ratio
ASR: Adjusted standardised residuals
AUC: Area under the receiver operating characteristic curve
Cox1: Cytochrome* c* oxidase subunit I
Cox2: Cytochrome* c* oxidase subunit II
FAD: Flea allergy dermatitis
IHMT: Instituto de Higiene e Medicina Tropical
LMA: Lisbon Metropolitan Area
NUTS-II: Nomenclature of Units for Territorial Statistics II
WAAVP: World Association for the Advancement of Veterinary Parasitology

## References

[1] Bitam I, Dittmar K, Parola P, Whiting MF, Raoult D. Fleas and flea-borne diseases. Int J Infect Dis. 2010;14:e667–76. 20189862 10.1016/j.ijid.2009.11.011

[2] Farkas R, Gyurkovszky M, Solymosi N, Beugnet F. Prevalence of flea infestation in dogs and cats in Hungary combined with a survey of owner awareness. Med Vet Entomol. 2009;23:187–94. 19712149 10.1111/j.1365-2915.2009.00798.x

[3] Cooper AR, Nixon E, Rose Vineer H, Abdullah S, Newbury H, et al. Fleas infesting cats and dogs in Great Britain: spatial distribution of infestation risk and its relation to treatment. Med Vet Entomol. 2020;34:452–8. 32697393 10.1111/mve.12462

[4] Bond R, Riddle A, Mottram L, Beugnet F, Stevenson R. Survey of flea infestation in dogs and cats in the United Kingdom during 2005. Vet Rec. 2007;160:503–6. 17435095 10.1136/vr.160.15.503

[5] Farrell S, McGarry J, Noble PJM, Pinchbeck GJ, Cantwell S, Radford AD, et al. Seasonality and other risk factors for fleas infestations in domestic dogs and cats. Med Vet Entomol. 2023;37:359–70. 36621899 10.1111/mve.12636 PMC10946788

[6] Rousseau J, Castro A, Novo T, Maia C. *Dipylidium caninum* in the twenty-first century: epidemiological studies and reported cases in companion animals and humans. Parasit Vectors. 2022;15:131. 35534908 10.1186/s13071-022-05243-5 PMC9088078

[7] Rust MK. The biology and ecology of cat fleas and advancements in their pest management: a review. Insects. 2017;8:118. 29077073 10.3390/insects8040118 PMC5746801

[8] León-Sosa A, Orlando SA, Mora-Jaramillo N, Calderón J, Rodriguez-Pazmino AS, Carvajal E, et al. First report of *Bartonella henselae* and *Bartonella clarridgeiae* carriage in stray cats from Ecuador and its link to a cat scratch disease outbreak in 2022. Acta Trop. 2024;257:107278. 38851625 10.1016/j.actatropica.2024.107278

[9] Abdullah S, Helps C, Tasker S, Newbury H, Wall R. Pathogens in fleas collected from cats and dogs: distribution and prevalence in the UK. Parasit Vectors. 2019;12:71. 30728050 10.1186/s13071-019-3326-x PMC6366081

[10] Iannino F, Sulli N, Maitino A, Pascucci I, Pampiglione G, Salucci S. Pulci di cane e gatto: Specie, biologia e malattie ad esse associate. Vet Ital. 2017;53:277–88. 29307121 10.12834/VetIt.109.303.3

[11] Self S, Yang Y, Walden H, Yabsley MJ, McMahan C, Herrin BH. A nowcast model to predict outdoor flea activity in real time for the contiguous United States. Parasit Vectors. 2024;17:27. 38254213 10.1186/s13071-023-06112-5 PMC10804753

[12] Pereira A, Cruz A, Novo T, Maia C. *Ctenocephalides felis* (cat flea). Trends Parasitol. 2025;41:249–50. 39818462 10.1016/j.pt.2024.12.016

[13] Lawrence AL, Webb CE, Clark NJ, Halajian A, Mihalca AD, Miret J, et al. Out-of-Africa, human-mediated dispersal of the common cat flea, *Ctenocephalides felis*: the hitchhiker’s guide to world domination. Int J Parasitol. 2019;49:321–36. 30858050 10.1016/j.ijpara.2019.01.001

[14] Zurita A, Trujillo I, García-Sánchez ÁM, Cutillas C. Survey of flea infestation in cats and dogs in Western Andalusia, Spain: seasonality and other risk factors for flea infestation. Med Vet Entomol. 2024;38:244–51. 38259177 10.1111/mve.12705

[15] Gálvez R, Montoya A, Checa R, Martín O, Marino V, Miró G. Flea species infesting dogs in Spain: updated spatial and seasonal distribution patterns. Med Vet Entomol. 2017;31:107–13. 27790728 10.1111/mve.12204

[16] Rinaldi L, Spera G, Musella V, Carbone S, Veneziano V, Iori A, et al. A survey of fleas on dogs in southern Italy. Vet Parasitol. 2007;148:375–8. 17683867 10.1016/j.vetpar.2007.06.036

[17] Koutinas AF, Papazahariadou MG, Rallis TS, Tzivara NH, Himonas CA. Flea species from dogs and cats in northern Greece: environmental and clinical implications. Vet Parasitol. 1995;58:109–15. 7676591 10.1016/0304-4017(94)00706-i

[18] Diakou A, Sofroniou D, Paoletti B, Tamvakis A, Kolencik S, Dimzas D, et al. Ticks, fleas, and harboured pathogens from dogs and cats in Cyprus. Pathogens. 2022;11:1403. 36558737 10.3390/pathogens11121403 PMC9786688

[19] Šlapeta J, King J, McDonell D, Malik R, Homer D, Hannan P, et al. The cat flea (*Ctenocephalides f. felis*) is the dominant flea on domestic dogs and cats in Australian veterinary practices. Vet Parasitol. 2011;180:383–8. 21515000 10.1016/j.vetpar.2011.03.035

[20] Beck HE, Zimmermann NE, McVicar TR, Vergopolan N, Berg A, Wood EF. Present and future Köppen-Geiger climate classification maps at 1-km resolution. Sci Data. 2018;5:180214. 30375988 10.1038/sdata.2018.214 PMC6207062

[21] Marchiondo AA, Holdsworth PA, Green P, Blagburn BL, Jacobs DE. World Association for the Advancement of Veterinary Parasitology (W.A.A.V.P) guidelines for evaluating the efficacy of parasiticides for the treatment, prevention and control of flea and tick infestation on dogs and cats. Vet Parasitol. 2007;145:332–44. 17140735 10.1016/j.vetpar.2006.10.028

[22] Linardi PM, Santos JLC.* Ctenocephalides felis felis* vs.* Ctenocephalides canis* (Siphonaptera: Pulicidae): some issues in correctly identify these species. Rev Bras Parasitol Vet. 2012;21:345–54. 23295817 10.1590/s1984-29612012000400002

[23] Beaucournu J-C, Launay H. Les Puces de France et du Bassin Méditerranéen Occidental. 1st ed. Paris: Fédération Française des Sociétés de Sciences Naturelles; 1990.

[24] Zurita A, Callejón R, De Rojas M, Cutillas C. Morphological, biometrical and molecular characterization of *Archaeopsylla erinacei* (Bouché, 1835). Bull Entomol Res. 2018;108:726–38. 29268804 10.1017/S0007485317001274

[25] Programa de Desenvolvimento Rural (PRODER). Freguesias por tipologia (Zonas Rurais e NUTS II). Revisão 2. 2014.Lisbon: Ministério da Agricultura. http://www.proder.pt/ResourcesUser/Documentos_Diversos/33/PDRc_Freg_ZRurais_NUTIIs_rev2_corrigido.pdf. Accessed 1 Jun 2025

[26] Whiting MF, Whiting AS, Hastriter MW, Dittmar K. A molecular phylogeny of fleas (Insecta: Siphonaptera): origins and host associations. Cladistics. 2008;24:677–707.

[27] Whiting MF, Ichael M, Hiting FW. Mecoptera is paraphyletic: multiple genes and phylogeny of Mecoptera and Siphonaptera. Zool Scr. 2002;31:93–104.

[28] Castresana J. Selection of conserved blocks from multiple alignments for their use in phylogenetic analysis. Mol Biol Evol. 2000;17:540–52. 10742046 10.1093/oxfordjournals.molbev.a026334

[29] Letunic I, Bork P. Interactive Tree of Life (iTOL) v6: recent updates to the phylogenetic tree display and annotation tool. Nucleic Acids Res. 2024;52:W78. 38613393 10.1093/nar/gkae268 PMC11223838

[30] Harvey ND. How old is my dog? identification of rational age groupings in pet dogs based upon normative age-linked process. Front Vet Sci. 2021;8:643085. 33987218 10.3389/fvets.2021.643085 PMC8110720

[31] Vogt AH, Rodan I, Brown M, Brown S, Buffington CAT, Forman MJL, et al. AAFP-AAHA: feline life stage guidelines. J Feline Med Surg. 2010;12:43–54. 20123486 10.1016/j.jfms.2009.12.006 PMC10845473

[32] Beck W, Boch K, Mackensen H, Wiegand B, Pfister K. Qualitative and quantitative observations on the flea population dynamics of dogs and cats in several areas of Germany. Vet Parasitol. 2006;137:130–6. 16442233 10.1016/j.vetpar.2005.12.021

[33] Moore CO, André MR, Šlapeta J, Breitschwerdt EB. Vector biology of the cat flea *Ctenocephalides felis*. Trends Parasitol. 2024;40:324–37. 38458883 10.1016/j.pt.2024.02.006 PMC11168582

[34] Gracia MJ, Calvete C, Estrada R, Castillo JA, Peribáñez MA, Lucientes J. Fleas parasitizing domestic dogs in Spain. Vet Parasitol. 2008;151:312–9. 18031934 10.1016/j.vetpar.2007.10.006

[35] Laudisoit A, Leirs H, Makundi RH, Van Dongen S, Davis S, Neerinckx S, et al. Plague and the human flea, Tanzania. Emerg Infect Dis. 2007;13:687. 17553245 10.3201/eid1305.061084 PMC2738476

[36] Zurita A, Callejón R, García-Sánchez M, Urdapilleta M, Lareschi M, Cutillas C. Origin, evolution, phylogeny and taxonomy of *Pulex irritans*. Med Vet Entomol. 2019;33:296–311. 30739354 10.1111/mve.12365

[37] Dobler G, Pfeffer M. Fleas as parasites of the family Canidae. Parasit Vectors. 2011;4:139. 21767354 10.1186/1756-3305-4-139 PMC3160944

[38] Lawrence AL, Brown GK, Peters B, Spielman DS, Morin-Adeline V, Šlapeta J. High phylogenetic diversity of the cat flea (*Ctenocephalides felis*) at two mitochondrial DNA markers. Med Vet Entomol. 2014;28:330–6. 24548270 10.1111/mve.12051

[39] Hornok S, Beck R, Farkas R, Grima A, Otranto D, Kontschán J, et al. High mitochondrial sequence divergence in synanthropic flea species (Insecta: Siphonaptera) from Europe and the Mediterranean. Parasit Vectors. 2018;11:221. 29609620 10.1186/s13071-018-2798-4 PMC5879554

[40] Miller W, Griffin C, Campbell K. Hypersensitivity disorders. In: Muller & Kirk’s Small Animal Dermatology. 7th ed. Amsterdam: Elsevier; 2013. p. 363–431.

[41] Kunkle GA, McCall CA, Stedman KE, Pilny A, Nicklin C, Logas DB. Pilot study to assess the effects of early flea exposure on the development of flea hypersensitivity in cats. J Feline Med Surg. 2003;5:287–94. 12948504 10.1016/S1098-612X(03)00026-3 PMC10822270

[42] Wilkerson MJ, Bagladi-Swanson M, Wheeler DW, Floyd-Hawkins K, Craig C, Lee KW, et al. The immunopathogenesis of flea allergy dermatitis in dogs, an experimental study. Vet Immunol Immunopathol. 2004;99:179–92. 15135984 10.1016/j.vetimm.2004.02.006

[43] Sperandei S. Understanding logistic regression analysis. Biochem Med. 2014;24:12. 10.11613/BM.2014.003 PMC3936971 24627710

[44] Cruz J, Belo-Pereira M, Fonseca A, Santos JA. Studies on heavy precipitation in Portugal: a systematic review. Climate. 2024;12:163.

[45] Metzger ME, Rust MK. Effect of temperature on cat flea (Siphonaptera: Pulicidae) development and overwintering. J Med Entomol. 1997;34:173–8. 9103760 10.1093/jmedent/34.2.173

[46] Pereira A, Martins Â, Brancal H, Vilhena H, Silva P, Pimenta P, et al. Parasitic zoonoses associated with dogs and cats: a survey of Portuguese pet owners’ awareness and deworming practices. Parasit Vectors. 2016;9:245. 27160667 10.1186/s13071-016-1533-2 PMC4862121

[47] Halos L, Beugnet F, Cardoso L, Farkas R, Franc M, Guillot J, et al. Flea control failure? Myths and realities. Trends Parasitol. 2014;30:228–33. 24661796 10.1016/j.pt.2014.02.007

