# Supplementary material for: Epidemiological and molecular characterisation of flea infestations in dogs and cats in mainland Portugal
Source: Parasit Vectors. 2025 Jul 6;18:263. doi: 10.1186/s13071-025-06904-x (PMC12229001; doi:10.1186/s13071-025-06904-x)
Supplement: Supplementary file 1 — Additional file 1. [file 13071_2025_6904_MOESM1_ESM.docx]

**Table S1.** Metadata of flea cytochrome oxidase subunit II (*cox2*) sequences included in the phylogenetic analysis.

| Species |  | Host species | Country | Accession number |
| --- | --- | --- | --- | --- |
| *Ctenocephalides. felis felis* |  | *Canis lupus familiaris* | Spain | LR991701 |
| *C. felis felis* |  | Colony | USA | NC049858 |
| *C. felis felis* |  | *Felis catus* | Kazakhstan | MF136071 |
| *C. felis felis* |  | *Felis catus* | Australia | HQ696926 |
| *C. felis felis* |  | *Felis catus* | Israel | MG637380 |
| *C. felis felis* |  | *Felis catus* | China | MW420044 |
| *C. felis felis* |  | *Felis catus* | Malta | MG637384 |
| *C. felis felis* |  | *Felis catus* | Italy | MG637383 |
| *C. felis felis* |  | *Canis lupus familiaris* | Croatia | MG637400 |
| *C. felis felis* |  | *Felis catus* | Hungary | MG637388 |
| *C. felis felis* |  | *Canis lupus familiaris* | AUSTRALIA | KF684916 |
| *C. felis felis* |  | *Vulpes vulpes* | Australia | KF684917 |
| *C. felis felis* |  | *Felis catus* | Australia | KF684914 |
| *C. felis felis* |  | *Canis lupus familiaris* | Fiji | KF684928 |
| *C. felis felis* |  | *Canis lupus familiaris* | Thailand | KF684922 |
| *C. felis felis* |  | Unknown | Senegal | JF966771 |
| *C. felis felis* |  | *Felis catus* | Israel | MG637379 |
| *C. felis felis* |  | *Felis catus* | Israel | MG637376 |
| *C. felis felis* |  | Environment | Australia | KF684920 |
| *C. felis felis* |  | *Canis lupus familiaris* | Fiji | KF684929 |
| *C. felis felis** |  | *Canis lupus familiaris* | Portugal | LC871646 |
| *C. felis felis** |  | *Canis lupus familiaris* | Portugal | LC871647 |
| *C. felis felis** |  | *Canis lupus familiaris* | Portugal | LC871648 |
| *C. felis felis** |  | *Canis lupus familiaris* | Portugal | LC871652 |
| *C. felis felis** |  | *Canis lupus familiaris* | Portugal | LC871655 |
| *C. felis felis** |  | *Canis lupus familiaris* | Portugal | LC871657 |
| *C. felis felis** |  | *Canis lupus familiaris* | Portugal | LC871658 |
| *C. felis felis** |  | *Canis lupus familiaris* | Portugal | LC871659 |
| *C. felis felis** |  | *Canis lupus familiaris* | Portugal | LC871660 |
| *C. felis felis** |  | *Canis lupus familiaris* | Portugal | LC871661 |
| *C. felis felis** |  | *Canis lupus familiaris* | Portugal | LC871665 |
| *C. felis felis** |  | *Canis lupus familiaris* | Portugal | LC871667 |
| *C. felis felis** |  | *Canis lupus familiaris* | Portugal | LC871669 |
| *C. felis felis** |  | *Canis lupus familiaris* | Portugal | LC871670 |
| *C. felis felis** |  | *Canis lupus familiaris* | Portugal | LC871672 |
| *C. felis felis** |  | *Canis lupus familiaris* | Portugal | LC871673 |
| *C. felis felis** |  | *Canis lupus familiaris* | Portugal | LC871674 |
| *C. felis felis** |  | *Canis lupus familiaris* | Portugal | LC871675 |
| *C. felis felis** |  | *Canis lupus familiaris* | Portugal | LC871676 |
| *C. felis felis** |  | *Canis lupus familiaris* | Portugal | LC871678 |
| *C. felis felis** |  | *Canis lupus familiaris* | Portugal | LC871679 |
| *C. felis felis** |  | *Canis lupus familiaris* | Portugal | LC871682 |
| *C. felis felis** |  | *Canis lupus familiaris* | Portugal | LC871683 |
| *C. felis felis** |  | *Canis lupus familiaris* | Portugal | LC871685 |
| *C. felis felis** |  | *Canis lupus familiaris* | Portugal | LC871686 |
| *C. felis felis** |  | *Canis lupus familiaris* | Portugal | LC871688 |
| *C. felis felis** |  | *Felis catus* | Portugal | LC871689 |
| *C. felis felis** |  | *Felis catus* | Portugal | LC871690 |
| *C. felis felis** |  | *Felis catus* | Portugal | LC871691 |
| *C. felis felis** |  | *Felis catus* | Portugal | LC871692 |
| *C. felis felis** |  | *Felis catus* | Portugal | LC871693 |
| *C. felis felis** |  | *Felis catus* | Portugal | LC871694 |
| *C. felis felis** |  | *Felis catus* | Portugal | LC871696 |
| *C. felis felis** |  | *Felis catus* | Portugal | LC871697 |
| *C. felis felis** |  | *Felis catus* | Portugal | LC871698 |
| *C. felis felis** |  | *Felis catus* | Portugal | LC871699 |
| *C. felis felis** |  | *Felis catus* | Portugal | LC871700 |
| *C. felis felis** |  | *Felis catus* | Portugal | LC871701 |
| *C. felis felis** |  | *Felis catus* | Portugal | LC871702 |
| *C. felis felis** |  | *Felis catus* | Portugal | LC871703 |
| *C. felis felis** |  | *Felis catus* | Portugal | LC871704 |
| *C. felis felis** |  | *Felis catus* | Portugal | LC871705 |
| *C. felis felis** |  | *Felis catus* | Portugal | LC871706 |
| *C. felis felis** |  | *Felis catus* | Portugal | LC871707 |
| *C. felis felis** |  | *Felis catus* | Portugal | LC871709 |
| *C. felis felis** |  | *Felis catus* | Portugal | LC871710 |
| *C. felis felis** |  | *Felis catus* | Portugal | LC871711 |
| *C. felis felis** |  | *Felis catus* | Portugal | LC871712 |
| *C. felis felis** |  | *Felis catus* | Portugal | LC871715 |
| *C. felis felis** |  | *Felis catus* | Portugal | LC871716 |
| *C. felis felis** |  | *Felis catus* | Portugal | LC871717 |
| *C. felis felis** |  | *Felis catus* | Portugal | LC871718 |
| *C. felis felis** |  | *Felis catus* | Portugal | LC871719 |
| *C. felis felis** |  | *Felis catus* | Portugal | LC871720 |
| *C. felis strongylus* |  | *Canis lupus familiaris* | Seychelles | KF684933 |
| *C. felis strongylus* |  | *Canis lupus familiaris* | Seychelles | KF684936 |
| *C. felis strongylus* |  | *Canis lupus familiaris* | Seychelles | KF684937 |
| *C. felis damarensis* |  | *Felis catus* | South Africa | MG586730 |
| *C. felis damarensis* |  | *Felis catus* | South Africa | MW074410 |
| *C. felis damarensis* |  | *Genetta tigrina* | South Africa | MW074408 |
| *C. felis damarensis* |  | *Genetta tigrina* | South Africa | MW074406 |
| *C. orientis* |  | *Canis lupus familiaris* | Thailand | KF684923 |
| *C. orientis* |  | *Canis lupus familiaris* | Thailand | KF684926 |
| *C. orientis* |  | *Canis lupus familiaris* | Thailand | KF684927 |
| *C. canis* |  | *Canis lupus familiaris* | Hungary | MG637369 |
| *C. canis* |  | cats/dogs | Czech Republic | MG586719 |
| *C. canis* |  | *Canis lupus familiaris* | China | NC_063710 |
| *C. canis* |  | *Canis lupus familiaris* | Iran | LR991705 |
| *C. canis* |  | *Canis lupus familiaris* | Croatia | MG637402 |
| *C. canis* |  | *Canis lupus familiaris* | Turkey | MG637403 |
| *C. canis** |  | *Canis lupus familiaris* | Portugal | LC871649 |
| *C. canis** |  | *Canis lupus familiaris* | Portugal | LC871651 |
| *C. canis** |  | *Canis lupus familiaris* | Portugal | LC871653 |
| *C. canis** |  | *Canis lupus familiaris* | Portugal | LC871654 |
| *C. canis** |  | *Canis lupus familiaris* | Portugal | LC871663 |
| *C. canis** |  | *Canis lupus familiaris* | Portugal | LC871666 |
| *C. canis** |  | *Canis lupus familiaris* | Portugal | LC871668 |
| *C. canis** |  | *Felis catus* | Portugal | LC871695 |
| *C. canis** |  | *Felis catus* | Portugal | LC871708 |
| *C. canis** |  | *Canis lupus familiaris* | Portugal | LC871713 |
| *Pulex irritans* |  | *Vormela peregusna* | China | MF136072 |
| *P. irritans* |  | *Canis lupus familiaris* | China | NC_063709 |
| *P. irritans* |  | Unknown | Spain | LR991748 |
| *P. irritans* |  | *Lycalopex griseus* | Argentina | LR991747 |
| *P. irritans* |  | *Homo sapiens* | Croatia | MG637397 |
| *P. irritans* |  | Small terrestrial mammals | Madagascar | KY073316 |
| *P. irritans* |  | *Meles meles* | Hungary | MG637395 |
| *P. irritans** |  | *Canis lupus familiaris* | Portugal | LC871650 |
| *P. irritans** |  | *Canis lupus familiaris* | Portugal | LC871656 |
| *P. irritans** |  | *Canis lupus familiaris* | Portugal | LC871662 |
| *P. irritans** |  | *Canis lupus familiaris* | Portugal | LC871664 |
| *P. irritans** |  | *Canis lupus familiaris* | Portugal | LC871671 |
| *P. irritans** |  | *Canis lupus familiaris* | Portugal | LC871677 |
| *P. irritans** |  | *Canis lupus familiaris* | Portugal | LC871680 |
| *P. irritans** |  | *Canis lupus familiaris* | Portugal | LC871681 |
| *P. irritans** |  | *Canis lupus familiaris* | Portugal | LC871684 |
| *P. irritans** |  | *Canis lupus familiaris* | Portugal | LC871687 |
| *Archaeopsylla erinacei* |  | *Erinaceus europaeus* | Portugal | MW114507 |
| *A. erinacei erinacei* |  | *Erinaceus europaeus* | France | LR991707 |
| *A. erinacei erinacei* |  | *Felis catus* | Hungary | MG637370 |
| *A. erinacei erinacei* |  | Unknown | Unknown | KM890861 |
| *A. erinacei maura* |  | *Erinaceus europaeus* | Spain | LR991706 |
| *A. erinacei maura** |  | *Canis lupus familiaris* | Portugal | LC871714 |
| *X. cheopis* |  | *Rattus norvegicus* | Malta | MG637385 |

*Sequences marked with an asterisk correspond to specimens collected during the present study.
