# Supplementary material for: Epidemiological and molecular characterisation of flea infestations in dogs and cats in mainland Portugal
Source: Parasit Vectors. 2025 Jul 6;18:263. doi: 10.1186/s13071-025-06904-x (PMC12229001; doi:10.1186/s13071-025-06904-x)
Supplement: Supplementary file 2 — Additional file 2. [file 13071_2025_6904_MOESM2_ESM.docx]

**Table S1** Prevalence of flea infestation in dogs treated with insecticidal compounds.

| Compounds | Examined, *n* (%) | Infested, *n* (%, 95% CI) | ASR |
| --- | --- | --- | --- |
| Afoxolaner | 50 (8.5) | 24 (48.0, 34.8-61.5) | -4.6 |
| Afoxalaner + Mylbemycin oxime | 5 (0.9) | 0 (0.0, 0.0-43.4) | -1.2 |
| Deltamethrin | 38 (6.5) | 14 (36.8, 23.4-52.7) | -2.2 |
| Dinotefuran + Permethrin + Pyriproxyfen | 48 (8.2) | 10 (20.8, 11.7-34.3) | -0.2 |
| Fipronil | 45 (7.7) | 26 (57.8, 43.3-71.0) | -6.0 |
| Fipronil + Imidacloprid + Permethrin | 1 (0.2) | 0 (0.0, 0.0-79.3) | -0.5 |
| Fipronil + Permethrin | 12 (2.0) | 2 (16.7, 4.7-44.8) | -0.5 |
| Flumethrin + Imidacloprid | 75 (12.8) | 7 (9.3, 4.6-18.0) | -1.2 |
| Fluralaner | 122 (20.8) | 13 (2.2, 6.317.4) | -3.4 |
| Imidacloprid | 1 (0.2) | 0 (0.0, 0.0-79.3) | -0.5 |
| Imidacloprid + Moxidectin | 2 (0.3) | 1 (50.0, 9.5-90.5) | -0.9 |
| Imidacloprid + Permethrin | 100 (17.1) | 26 (4.4, 18.4-35.4) | -1.0 |
| Indoxacarb | 3 (0.5) | 0 (0.0, 0.0-56.1) | -0.9 |
| Indoxacarb + Permethrin | 6 (1.0) | 0 (0.0, 0.0-39.0) | -1.3 |
| Indoxacarb + Sarolaner | 1 (0.2) | 0 (0.0, 0.0-79.3) | -0.5 |
| Lotilaner | 15 (2.6) | 0 (0.0, 0.0-20.4) | -2.1 |
| Moxidectin + Sarolaner | 2 (0.3) | 0 (0.0, 0.0-65.8) | -0.8 |
| Permethrin | 7 (1.2) | 1 (14.3, 2.6-51.3) | -0.5 |
| Sarolaner | 49 (8.4) | 5 (10.2, 4.4-21.8) | -2.1 |
| Selamectin | 3 (0.5) | 0 (0.0, 0.0-56.1) | -0.9 |
| Spinosad | 1 (0.2) | 1 (1.9, 20.7-100.0) | -1.9 |
| *P*-value < 0.0001, derived from the Fisher-Freeman-Halton test.  ASR: Adjusted standardized residuals; CI: Confidence interval. | | | |

**Table S2** Prevalence of flea infestation in cats treated with insecticidal compounds.

| Compounds | Examined, *n* (%) | Infested, *n* (%, 95% CI) | ASR |
| --- | --- | --- | --- |
| Afoxolaner | 6 (1.1) | 0 (0.0, 0.0-39.0) | -1.5 |
| Dinotefuran + Fipronil + Fluralaner + Imidacloprid + Moxidectin + Pyriproxyfen + (S)-Methoprene | 1 (0.2) | 0 (0.0, 0.0-79.3) | -0.6 |
| Dinotefuran + Permethrin + Pyriproxyfen | 17 (3.2) | 3 (17.6, 6.2-41.0) | -0.8 |
| Dinotefuran + Pyriproxyfen | 16 (3.0) | 3 (18.8, 6.6-43.0) | -0.7 |
| Eprinomectin + Esafoxolaner | 31 (5.8) | 3 (9.7, 3.3-24.9) | -2.1 |
| Eprinomectin + Fipronil | 60 (11.2) | 3 (5.0, 1.7-13.7) | -3.9 |
| Eprinomectin + Fipronil + Pyriproxyfen + (S)-Methoprene | 1 (0.2) | 1 (100.0, 20.7-100.0) | -1.7 |
| Fipronil | 107 (20.0) | 63 (58.9, 49.4-67.7) | -8.7 |
| Fipronil + (S)-Methoprene | 13 (2.4) | 9 (69.2, 42.4-87.3) | -3.6 |
| Fipronil + Imidacloprid | 1 (0.2) | 0 (0.0, 0.0-79.3) | -0.6 |
| Fipronil + Permethrin | 1 (0.2) | 1 (100.0, 20.7-100.0) | -1.7 |
| Fipronil + Pyriproxyfen | 8 (1.5) | 8 (100.0, 67.6-100.0) | -4.8 |
| Flualaner + Flumethrin + Imidacloprid + Moxidectin | 1 (0.2) | 1 (100.0, 20.7-100.0) | -1.7 |
| Flumethrin + Imidacloprid | 19 (3.6) | 0 (0.0, 0.0-16.8) | -0.6 |
| Fluralaner | 52 (9.7) | 11 (21.2, 12.2-34.0) | -0.8 |
| Fluralaner + Imidacloprid + Moxidectin | 1 (0.2) | 0 (0.0, 0.0-79.3) | -0.6 |
| Fluralaner + Moxidectin | 34 (6.4)) | 0 (0.0, 0.0-10.2) | -3.6 |
| Imidacloprid | 33 (6.2) | 6 (18.2, 8.6-34.4) | -1.1 |
| Imidacloprid + Lotilaner | 1 (0.2) | 0 (0.0, 0.0-79.3) | -0.6 |
| Imidacloprid + Moxidectin | 28 (5.2) | 4 (14.3, 5.7-31.5) | -1.4 |
| Imidacloprid + Moxidectin + Sarolaner + Selamectin | 1 (0.2) | 0 (0.0, 0.0-65.8) | -0.6 |
| Imidacloprid + Permethrin | 2 (0.4) | 1 (50.0, 9.5-90.5) | -0.8 |
| Lotilaner | 37 (6.9) | 12 (32.4, 19.6-48.5) | -0.9 |
| Lotilaner + Milbemycin | 1 (0.2) | 0 (0.0, 0.0-79.3) | -0.6 |
| Lotilaner + Sarolaner + Selamectin | 1 (0.2) | 0 (0.0, 0.0-79.3) | -0.6 |
| Milbemycin oxime | 1 (0.2) | 0 (0.0, 0.0-79.3) | -0.6 |
| Moxidectin + Sarolaner | 1 (0.2) | 0 (0.0, 0.0-79.3) | -0.6 |
| Nitenpyram | 1 (0.2) | 0 (0.0, 0.0-79.3) | -0.6 |
| Sarolaner | 6 (1.1) | 0 (0.0, 3.0-56.4) | -1.5 |
| Sarolaner + Selamectin | 7 (1.3) | 0 (0.0, 0.0-35.4) | -1.6 |
| Selamectin | 46 (8.6) | 10 (21.7, 12.3-35.6) | -0.7 |
| *P*-value < 0.0001, derived from the Fisher-Freeman-Halton test.  ASR: Adjusted standardized residuals; CI: Confidence interval. | | | |

**Table S3** Potential risk factors for flea infestation in dogs in mainland Portugal.

| Variable (Reference category) | *β* | *SE* | *χ^2^_Wald_* | *df* | *P*-value | aOR (95% CI) |
| --- | --- | --- | --- | --- | --- | --- |
| Region - NUTS II (North) |  |  |  |  |  |  |
| Center | -0.10 | 0.25 | 0.15 | 1 | 0.699 | 0.91 (0.55-1.49) |
| Lisbon Metropolitan Area | -1.95 | 0.35 | 31.85 | 1 | <0.0001* | 0.14 (0.07-0.28) |
| Alentejo | -0.97 | 0.30 | 10.35 | 1 | 0.001* | 0.38 (0.21-0.69) |
| Algarve | -0.79 | 0.27 | 8.81 | 1 | 0.003* | 0.45 (0.27-0.74) |
| Season (Winter) |  |  |  |  |  |  |
| Spring | 0.73 | 0.34 | 4.60 | 1 | 0.032* | 2.08 (1.07-4.06) |
| Summer | 1.04 | 0.35 | 8.88 | 1 | 0.003* | 2.83 (1.43-5.61) |
| Autumn | 1.31 | 0.35 | 13.85 | 1 | <0.0001* | 3.72 (1.86-7.43) |
| No use of Insecticides | 1.58 | 0.30 | 27.57 | 1 | <0.0001* | 4.87 (2.70-8.79) |
| Constant | -1.59 | 0.32 | 25.02 | 1 | <0.0001* |  |
| Homer and Lemeshow test: *χ2* = 7.96, *df* = 8, *P* = 0.438; Likelihood ratio test (*G²*) = 85.22, *df* = 8, *P* < 0.0001; Area under the curve = 0.71, *P* < 0.0001. | | | | | | |
| *Statistically significant at *α* = 0.05.  aOR: adjusted odds ratio; CI: Confidence interval; NUTS: Nomenclature of units for territorial statistics. | | | | | | |

**Table S4** Potential risk factors for flea infestation in cats in mainland Portugal.

| Variable (Reference category) | *β* | *SE* | *χ^2^_Wald_* | *df* | *P*-value | aOR (95% CI) |
| --- | --- | --- | --- | --- | --- | --- |
| Region - NUTS II (North) |  |  |  |  |  |  |
| Center | -0.81 | 0.30 | 7.08 | 1 | 0.008* | 0.45 (0.25-0.81) |
| Lisbon Metropolitan Area | -2.30 | 0.46 | 24.73 | 1 | < 0.0001* | 0.10 (0.04-0.25) |
| Alentejo | -1.26 | 0.40 | 10.08 | 1 | 0.001* | 0.28 (0.13-0.62) |
| Algarve | -0.62 | 0.33 | 3.49 | 1 | 0.062 | 0.54 (0.28-1.03) |
| Lifestyle (Domestic outdoor) |  |  |  |  |  |  |
| Sheltered | -3.52 | 0.56 | 39.82 | 1 | < 0.0001* | 0.03 (0.10-0.09) |
| Stray | -2.35 | 0.67 | 12.30 | 1 | < 0.0001* | 0.10 (0.03-0.36) |
| Domestic indoor | -2.64 | 0.51 | 26.73 | 1 | < 0.0001* | 0.07 (0.03-0.19) |
| Domestic indoor-outdoor | -1.67 | 0.49 | 11.62 | 1 | 0.001* | 0.19 (0.07-0.49) |
| No use of Insecticides | 1.39 | 0.33 | 17.88 | 1 | < 0.0001* | 4.02 (2.11-7.67) |
| Constant | 1.61 | 0.49 | 10.69 | 1 | 0.001 | 4.99 |
| Homer and Lemeshow test: *χ2* = 9.72, *df* = 7, *P* = 0.205; Likelihood ratio test (*G²*) = 136.12, *df* = 9, *P* < 0.0001; Area under the curve= 0.77, *P* = < 0.0001. | | | | | | |
| *Statistically significant at *α* = 0.05.  aOR: adjusted odds ratio; CI: Confidence interval; NUTS: Nomenclature of units for territorial statistics. | | | | | | |
